# Overexpression of soybean trypsin inhibitor genes decreases defoliation by corn earworm (*Helicoverpa zea*) in soybean (*Glycine max*) and *Arabidopsis thaliana*

**DOI:** 10.3389/fpls.2023.1129454

**Published:** 2023-02-17

**Authors:** Mst Shamira Sultana, Mitra Mazarei, Juan Luis Jurat-Fuentes, Tarek Hewezi, Reginald J. Millwood, C. Neal Stewart

**Affiliations:** ^1^ Department of Plant Sciences, University of Tennessee, Knoxville, TN, United States; ^2^ Center for Agricultural Synthetic Biology, University of Tennessee, Knoxville, TN, United States; ^3^ Department of Entomology and Plant Pathology, University of Tennessee, Knoxville, TN, United States

**Keywords:** trypsin inhibitors, overexpression, tissue-specific promoter, transgenic soybean and Arabidopsis, trypsin and chymotrypsin enzymes inhibition, corn earworm, soybean cyst nematode

## Abstract

Trypsin inhibitors (TIs) are widely distributed in plants and are known to play a protective role against herbivores. TIs reduce the biological activity of trypsin, an enzyme involved in the breakdown of many different proteins, by inhibiting the activation and catalytic reactions of proteins. Soybean (*Glycine max*) contains two major TI classes: Kunitz trypsin inhibitor (KTI) and Bowman-Birk inhibitor (BBI). Both genes encoding TI inactivate trypsin and chymotrypsin enzymes, which are the main digestive enzymes in the gut fluids of Lepidopteran larvae feeding on soybean. In this study, the possible role of soybean TIs in plant defense against insects and nematodes was investigated. A total of six TIs were tested, including three known soybean trypsin inhibitors (KTI1, KTI2 and KTI3) and three genes encoding novel inhibitors identified in soybean (KTI5, KTI7, and BBI5). Their functional role was further examined by overexpression of the individual TI genes in soybean and Arabidopsis. The endogenous expression patterns of these TI genes varied among soybean tissues, including leaf, stem, seed, and root. *In vitro* enzyme inhibitory assays showed significant increase in trypsin and chymotrypsin inhibitory activities in both transgenic soybean and Arabidopsis. Detached leaf-punch feeding bioassays detected significant reduction in corn earworm (*Helicoverpa zea*) larval weight when larvae fed on transgenic soybean and Arabidopsis lines, with the greatest reduction observed in KTI7 and BBI5 overexpressing lines. Whole soybean plant greenhouse feeding bioassays with *H. zea* on KTI7 and BBI5 overexpressing lines resulted in significantly reduced leaf defoliation compared to non-transgenic plants. However, bioassays of KTI7 and BBI5 overexpressing lines with soybean cyst nematode (SCN, *Heterodera glycines*) showed no differences in SCN female index between transgenic and non-transgenic control plants. There were no significant differences in growth and productivity between transgenic and non-transgenic plants grown in the absence of herbivores to full maturity under greenhouse conditions. The present study provides further insight into the potential applications of TI genes for insect resistance improvement in plants.

## Introduction

Soybean (*Glycine max*) is an economically important crop worldwide. Yield loss resulting from a variety of herbivorous insects and pests is invariably a concern for farmers. Lepidopteran larvae, including the soybean looper (*Chrysodeixis includens*), the velvet bean caterpillar (*Anticarsia gemmatalis*), the fall armyworm (*Spodoptera* spp.), and the corn earworm (*Helicoverpa zea*) are major defoliators that cause soybean yield losses (Horikoshi et al., 2021; Thrash et al., 2021). In 2017, US economic losses and cost of *H. zea* control in soybean represented more than $160 million (Musser et al., 2018). Root pests, such as the soybean cyst nematode (SCN; *Heterodera glycines*) commonly cause significant yield losses in soybean (Bradley et al., 2021). To protect themselves, plants have their own specific defense responses *via* toxin production as well as other defensive-related proteins (Stout et al., 1997; Walling, 2000; De Vos et al., 2005; Howe and Jander, 2008; Alba et al., 2011). These responses can affect herbivores growth and development, feeding, fertility, and fecundity (Broadway, 1996; Srinivasan et al., 2005; Kuwar et al., 2020).

The application of chemical pesticides and the widespread adoption of transgenic soybeans engineered to express *Bacillus thuringiensis* (Bt) toxins are commonly used to control herbivorous insects and pests (Stewart et al., 1996; Walker et al., 2004; Panizzi, 2013; James, 2015; Machado et al., 2020). Both approaches carry concerns such as residual pesticides found in soil and water, and the negative effects on non-target organisms (Quilis et al., 2014; Mahmood et al., 2016) and the potential for evolution of Bt resistance in target insect species (Tabashnik et al., 2009; Carriere et al., 2010), that require alternative management practices for Bt soybean. To address these issues, plant biotechnology has offered several new defense strategies against insect pests that include RNA interference (RNAi) (Baum et al., 2007; Kim et al., 2015; Tian et al., 2015), plastid transformation technology such as multiple gene stacking and high transgene expression levels (Dufourmantel et al., 2004; Valkov et al., 2011; Chen et al., 2014), and the overexpression of defense-related genes (Sunkar et al., 2003; Vannini et al., 2004; Christou et al., 2006; Kiggundu et al., 2010), that have the potential to yield improved crop varieties with enhanced insect pest resistance.

Among native plant defenses, proteinase inhibitors (PIs) have direct effects on herbivores by interfering with protein digestion in their gut (Green and Ryan, 1972; Santamaria et al., 2015; Martinez et al., 2016; Alfonso-Rub´ et al., 2003; Tamhane et al., 2005; Telang et al., 2009; Chen et al., 2014; Grosse-Holz and van der Hoorn, 2016; Arnaiz et al., 2018). PIs are widely present in plant storage organs (seeds and tubers) and are known to inhibit plant pests (Ryan, 1990; Koiwa et al., 1997; Lee et al., 1999). PIs including serine, cysteine, and aspartic, are classified according to their active site group (Rawlings et al., 2004; Rawlings et al., 2010). Most lepidopteran digestive systems are largely based on serine proteinases (trypsin and chymotrypsin), which can be deactivated by plant serine PIs (Srinivasan et al., 2006; Shamsi et al., 2016; Terra and Ferreira, 2020). The utilization of these inherent defensive genes in a genetic engineering strategy may inhibit insect herbivory through changes in expression patterns or through PI overexpression in leaves (Sultana et al., 2022). PI expression in leaves could prevent insect damage and eventual yield loss.

Soybean trypsin inhibitors (TIs) are the most widely studied serine family members of plant PIs. Based on their cysteine-residue content and the number of protein binding sites soybean TIs are grouped into Kunitz trypsin inhibitors (KTIs), and Bowman-Birk inhibitors (BBIs) (Laskowski and Kato, 1980). The KTIs can have single or double polypeptide chains and contain four cysteine residues that form two disulfide bridges, with a single reactive site. BBIs are composed of single-chain polypeptides with fourteen cysteine residues that form seven disulfide bridges, and two reactive sites. The structural pattern of KTI and BBI inhibitors allows them to interact with and inhibit insect serine proteinases (Sultana et al., 2022). KTIs and BBIs are commonly found in legume seeds and are known as defensive proteins (Macedo et al., 2004; Azzouz et al., 2005; Srinivasan et al., 2005; Müller et al., 2017; Sultana et al., 2022).

The utilization of these inherent defensive genes for crop protection can reduce yield loss. Plant TIs have negative effects on the digestive system of herbivores and soybean seeds are known to accumulate high amounts of inherent anti-digestive TIs (Ryan, 1990; Birk, 2003). Previous studies suggest that overexpression of TI genes can confer insect resistance to rice (Lee et al., 1999; Vila et al., 2005; Quilis et al., 2014; Zhou et al., 2017), tobacco (Koo et al., 1992; Tissot et al., 2008; Chen et al., 2014; Turrà et al., 2020), Arabidopsis (Quain et al., 2014; Malefo et al., 2020), potato (Marchetti et al., 2000; Outchkourov et al., 2004), cotton (Franco et al., 2004; Ni et al., 2017; Ibrahim et al., 2020), sugarcane (Falco and Silva-Filho, 2003), and white clover (McManus et al., 2005). In addition, TI-expressing transgenic plants have shown increased tolerance to drought stress (Dramé et al., 2013; Kidrič et al., 2014). Furthermore, the co-expression of two different TI has also shown higher insecticidal activity in a broader range of insects (Santamaria et al., 2012; Senthilkumar et al., 2010; Cingel et al., 2017). Co-expression of TI genes and Bt toxin were also found to enhance plant resistance to insect herbivory (Li et al., 2000; Qiu, 2008; Deka and Barthakur, 2010). The generation of TI mutants by site-directed mutagenesis could potentially led on insect resistance (Kiggundu et al., 2006; Goulet et al., 2008). Synthetic biology may someday be used to create novel TI proteins with multiple target inhibition properties, i.e., different proteinases in multiple pest species (Sultana et al., 2022). Altogether, these findings suggest that TI genes have the potential to enhance herbivore resistance.

In molecular genetics, the promoter is an important *cis*-regulatory element that regulates gene transcription. In many genetic engineering approaches constitutive promoters are often used to drive transgene expression. Although this promoter type may lead to high expression levels, transgene expression may not be necessary in all plant tissues. In this case tissue-specific promoters may be a better choice. For example, tissue-specific promoters can be used to direct TI expression to the tissue-of-interest (such as leaves and stems) and restrict expression in tissues where TI expression is unnecessary (such as roots). Furthermore, green tissue-specific promoters can direct transgene expression in shoots with negligible to no expression in roots. Using this approach for TI synthesis in leaves could be an effective strategy to provide resistance against foliar insects.

To examine this approach, *Arabidopsis thaliana* and soybean (*G. max*) plants were engineered with six individual soybean TI genes under the control of either a constitutive promoter *Cauliflower mosaic virus* 35S (CaMV 35S) or a green tissue-specific promoter ribulose-1,5-bisphosphate carboxylase small subunit gene SRS4 (rbcs-SRS4). Transgenic plants were evaluated for resistance against corn earworm larvae and SCN. Furthermore, TI genes were compared to determine those with the highest efficiency against insect herbivory. The results presented here provide insight into the potential use of TI genes as a strategy for insect resistance in genetically engineered plants.

## Materials and methods

### Expression vector constructions

Based on endogenous gene expression, five soybean Kunitz trypsin inhibitor (KTI) genes KTI1 (Glyma.01G095000.1), KTI2 (Glyma.09G155500.1), KTI3 (Glyma.08G341500.1), KTI5 (Glyma.08G341700.1), KTI7 (Glyma.08G341300.1) and one Bowman-Birk inhibitor gene BBI5 (Glyma.01G096200.1) were selected for overexpression studies. The coding region corresponding to each of these six genes was amplified by PCR from soybean cv. ‘Williams 82’ using primers incorporating specific restriction enzymes (*Spe*I and *Asc*I) (**Supplementary Table S1**). The amplified gene products were purified using a DNA Clean & Concentrator kit (Zymo Research, Irvine, CA, USA) according to the manufacturer’s protocol, and then digested with restriction enzymes. The binary vector pB2GW7 (Karimi et al., 2002) was used to generate the individual TI gene construct. The pB2GW7 binary vector consists of a Nos promoter*::*Bar*::*Nos terminator cassette for plant selection. The vector pB2GW7 was also digested with *Spe*I and *Asc*I restriction enzymes and purified using the DNA Clean & Concentrator kit (Zymo Research). After purification of the vector and gene products, ligation reactions were performed to clone the individual TI gene downstream of the CaMV 35S promoter (**Supplemental Figure S1**) followed by the transformation in *E. coli* with spectinomycin as bacterial selection. Each soybean TI gene was also cloned under the control of a green tissue-specific promoter. Soybean green-tissue specific promoter (1775 bp) of ribulose-1,5-bisphosphate carboxylase small subunit gene SRS4 (rbcs-SRS4) (Cui et al., 2015) was amplified by PCR from soybean cv. ‘Williams 82’ using primers incorporating specific restriction enzymes (*Sac*I and *Spe*I) (**Supplementary Table S1**). The amplified promoter fragment was purified and cloned upstream of the TI gene *via* restriction enzymes (*Sac*I and *Spe*I) in the binary vector of pB2GW7 (**Supplementary Figure S1**). The insertions were confirmed by sequencing. Two types of constructs were created for each TI gene. One set was produced using the CaMV 35S promoter and the other set utilized the rbcS-SRS4 promoter. All constructs were individually transferred into *Agrobacterium tumefaciens* strain EHA101 *via* the heat-shock method. The insertion into *Agrobacterium* was confirmed by colony PCR using the gene-specific primer sets. The primer sequences used in this study are listed in **Supplementary Table S1**.

### KTIs and BBI protein sequence analysis

Protein sequence of KTI1, KTI2, KTI3, KTI5, KTI7 and BBI5 were obtained from Phytozome database v13 (https://phytozome-next.jgi.doe.gov/). Protein sequence alignments (**Supplemental Figure S1C**) were performed in Clustal Omega (https://www.ebi.ac.uk/Tools/msa/clustalo/).

### *Nicotiana benthamiana* transient transformation assays

To evaluate transient TI gene expression, *A. tumefaciens* containing individual CaMV 35S-TI constructs were used in *N. benthamiana* agroinfiltration experiments. *Agrobacterium*-containing individual gene constructs were added to YEP media (5 g/L NaCl, 10 g/L peptone, and 10 g/L yeast extract) with 50 mg/L kanamycin and 200 mg/L spectinomycin and grown overnight in a sterile flask placed in an incubating shaker set at 28 °C and 225 rpm. Acetosyringone (100 µM) was added to the culture for 1 h before removing the flasks from the shaking platform. Cultures were centrifuged at 3000 × g for 15 min. The pelleted cells were resuspended with infiltration buffer (10 mM MgCl2, 10 mM MES, 100 µM acetosyringone, pH 5.6) to an optical density OD_600_ of 0.5. *Agrobacterium* solution was incubated in the dark at room temperature for 3 h before infiltration. Four-week-old *N. benthamiana* plants were grown in a growth chamber (Percival Scientific Inc. Perry, IA, USA) at a day/night temperature of 23 °C, 300 µmol/m^2^ s light intensity, and photoperiod of 16/8 h light/dark cycle for vacuum infiltration. The plants were immersed in the *Agrobacterium* solution and placed into a 20 L vacuum chamber (Best Value Vacs, Naperville, IL, USA). Vacuum pressure of approximately -84 kPa was applied three times for 1 min. For mock control treatments, a 10 mM MgCl2 solution was used for the vacuum infiltration of plants. After infiltration, filter paper was used to remove the excess bacterial solution. The infiltrated plants were covered with a clear plastic lid and returned to the growth chamber. Six independent biological replicates were used for each gene construct.

### Generation of transgenic Arabidopsis plants

*A. tumefaciens* containing individual CaMV 35S-TI constructs was used for Arabidopsis transformation. Wild-type *Arabidopsis thaliana* (ecotype Col-0) plants were grown in the growth chamber at a day/night temperature of 24 °C under a photoperiod of 16/8 h (light/dark) with 140 µmol/m^2^ s light intensity. Eight-week-old Arabidopsis plants were used for transformation. Primary inflorescence buds were clipped to allow the formation of multiple inflorescence buds. *A. thaliana* transformation was individually carried out for each CaMV 35S-TI construct *via* the *Agrobacterium* floral dip method (Zhang et al., 2006). Briefly, multiple inflorescences were submerged in the *Agrobacterium* solution for 10 s, and then were allowed to grow in the growth chamber until seed maturity. Three independently transformed plants were selected for each CaMV 35S-TI construct. T_1_ generation seeds from individual T_0_ plants were harvested and sterilized in 70% ethanol for 1 min, washed with sterile water followed by 50% bleach for 8 min then washed with sterile water for seven times. Sterilized seeds were plated on half-strength MS-medium containing 6 mg/L glufosinate-ammonium. On each plate, 50 seeds were germinated. The transgenic lines were based on single copy lines, with a segregation ratio of 3:1 (resistant: susceptible) in the T_2_ generation, and homozygous lines were presumed if there was no segregation in the T_3_ generation (n > 100). Selected seedlings were transplanted into the pot containing potting mix (Sun Gro Horticulture, Agawam, MA, USA) for further growth. Transgenic Arabidopsis were allowed to self and homozygous T_3_ seeds were selected for further experiments.

### Generation of stable transgenic soybean plants

Strains of *A. tumefaciens* containing individual CaMV 35S-TI constructs or individual rbcS-SRS4-TI constructs were used for soybean cv. ‘TN15-5007’ transformation. The binary constructs were introduced into soybean cotyledons by *Agrobacterium-*mediated transformation (Li et al., 2017). All explants were cultured in a growth chamber at 24 °C (light/dark) under a photoperiod of 16/8 h (light/dark) with 140 µmol/m^2^ s light intensity. Shoots were generated on a selective medium containing 6 mg/L glufosinate-ammonium. After rooting, putative transgenic plantlets were transferred to potting mix (Sun Gro Horticulture, Agawam, MA, USA). Transgenic T_0_ soybean plants were confirmed for each construct by painting Finale^®^ herbicide with glufosinate-ammonium as an active ingredient (Bayer CropScience, Research Triangle Park, NC, USA) on the upper surface of the leaf (Paz et al., 2006). Wild-type soybean leaf was also painted as a negative control. Transgenic T_1_ progeny of the individual lines was also confirmed using Finale^®^ herbicide with a segregation ratio of 3:1 (resistant: susceptible). T_2_ seeds were harvested from self-pollinated T_1_ progeny. T_2_ progeny were screened for herbicide selection and those that showed 100% resistance to Finale^®^ herbicide were selected. Independent homozygous T_3_ lines for each promoter construct were selected for further analysis. A chi-squared test was conducted to determine whether observed segregation ratios were significantly different from expected ratios.

### Molecular analysis of transgenic Arabidopsis and soybean plants

Total genomic DNA was isolated from 1 g of fresh leaves of three-week-old Arabidopsis and soybean plants using the CTAB extraction method (Stewart and Via, 1993). The insertion of the transgene (Nos promoter driving *Bar* gene) and (2×35S promoter driving TI genes), and (rbcS-SRS4 promoter driving TI genes) was confirmed by PCR using T_3_ transgenic Arabidopsis and soybean genomic DNA as a template (**Supplemental Figures S2–S4**). Genomic DNA was diluted to 100 ng/µL for PCR. PCR conditions for the *Bar* gene were as follows: 98°C for 2 min followed by 30 cycles at 98°C for 30 s, 64°C for 30 s, and 72°C for 30 s, and a final extension at 72°C for 5 min. PCR conditions for the TI genes were as follows: 98°C for 2 min followed by 30 cycles at 98°C for 30 s, 60°C for 30 s, and 72°C for 30 s, and a final extension at 72°C for 5 min. Testing for *Agrobacterium* contamination in transgenic lines was performed *via* PCR using a primer set from the *Agrobacterium* backbone. To amplify the *Agrobacterium chvA* (chromosomal virulence gene A) gene, as a control for the *Agrobacterium* contamination, the PCR conditions were as follows: 98°C for 2 min followed by 30 cycles at 98°C for 30 s, 65°C for 30 s, and 72°C for 30 s, and a final extension at 72°C for 5 min (**Supplemental Figures S2B, S3B, S4B**). PCR products were visualized on 0.8% agarose gels containing ethidium bromide. Primers used for the genotypic analysis of transgenic lines are provided in the **Supplementary Table S2, S3**.

### *In vitro* enzymatic assay of transgenic Arabidopsis and soybean plants

The trypsin inhibition analysis was performed as described by Malefo et al. (2020). Total protein was extracted from six-week-old T_3_ transgenic and non-transgenic Arabidopsis and soybean plants for each gene construct. Arabidopsis rosettes or soybean leaves were ground and resuspended in protein extraction buffer (0.15 M NaCl, 50 mM sodium phosphate pH 6.2, and 2 mM EDTA pH 8.0) for 1 h at 4 °C. The content was centrifuged at 8000 g for 15 min and the supernatant was transferred to fresh tubes. Total protein content was determined using the Qubit Protein Assay Kit (Thermo Fisher Scientific, Pittsburgh, PA, USA). *In vitro* inhibitory activity of transgenic plant protein was tested against commercial bovine TPCK-treated trypsin (EC 3.4.21.4) (≥10,000 BAEE units/mg, Millipore Sigma, Saint Louis, MO, USA) and bovine TLCK-treated chymotrypsin (EC 3.4.21.1) ((≥40 units/mg, Millipore Sigma). Specific activities were assayed with the synthetic substrate; Nα-Benzoyl-DL-arginine 4-nitroanilide hydrochloride (BApNA) (Millipore Sigma) for trypsin and N-Succinyl-Ala-Ala-Pro-Phe p-nitroanilide (SAAPFpNA) (Millipore Sigma) for chymotrypsin. Enzyme activity was performed in 0.2 mL reactions in 96-well black non-stick microtiter plates. Protein extracts (20 µg) from transgenic and non-transgenic control lines were incubated with 100 ng of commercial trypsin in buffer (50 mM Tris-HCl, pH 8.2) for 10 min, and then the substrate was added to a 0.2 mM final concentration and incubated for 1 h at 28 °C. The absorbance of the reaction product, ρ-nitroaniline, was measured at 405 nm using a Synergy H1 multi-detection microplate reader (Bio-Tek Instruments Inc., Santa Clara, CA, USA). A standard curve was generated using set quantities of soybean trypsin inhibitor (Millipore Sigma) or chymotrypsin inhibitor (Millipore Sigma). Inhibitory activity was calculated and expressed as a percentage of inhibition of transgenic protein relative to that wild-type protein. Three independent transgenic lines were used for each gene construct and six replicate plants were used for each line. All assays were carried out in triplicate for each plant.

### Detached leaf feeding bioassays

The effect of each TI gene overexpression on insect leaf defoliation was evaluated in transgenic Arabidopsis and soybean lines through detached leaf punch bioassays (Arnaiz et al., 2018) with corn earworm (*H. zea*) larvae. Corn earworm eggs were purchased from Benzon Research (Carlisle, PA, USA) and hatched in the growth chamber at 26 °C on a 16/8 h (light/dark) photoperiod, light intensity (150 µmol/m^2^ s). Leaves were removed from six-week-old transgenic Arabidopsis or soybean plants and placed in a plastic bag with water spray to keep moistened for a few hours. Leaf punches (1 cm^2^) were cut and transferred to individual cells of 128-cell plastic bioassay trays (Frontiers Agricultural Sciences, Newark, DE, USA). Each cell was filled with 1 mL of 2% agarose solution to preserve turgidity of the leaf punch. Six-week-old wild-type plant leaf punches were used as a control. A single 1^st^ instar was tested per cell. During the assay, the old leaf punches were replaced with fresh leaf punches every three days. Three independent transgenic lines were used for each gene construct and six replicate plants were used for each line. Sixteen cells were filled with leaf punches from a single plant. All bioassay trays were kept in the same conditions as above mentioned. Larval weight was recorded after eight days of feeding.

### Whole plant feeding bioassays

Bioassays on transgenic soybean plants were performed with plants grown in a polyester-mesh cage under greenhouse conditions. The environmental conditions of the greenhouse were 16/8 h (light/dark) photoperiod and 25 °C temperature with fluctuations from a minimum of 22 °C to a maximum of 28 °C. In whole plant feeding bioassays, plants overexpressing the two best-performed genes (KTI7 and BBI5) were selected based on the results from detached leaf feeding bioassays for CaMV 35S-TI or rbcS-SRS4-TI. The mesh cage contained six-week-old T_3_ transgenic as well as wild-type control plants. Three independent transgenic lines for each gene and six replicate plants per line were used in the experiments. Corn earworm eggs were hatched and larvae reared on artificial diet (beet armyworm diet, Frontiers Agricultural Sciences, Newark, DE, USA) for four days in a growth chamber at 26 °C on a 16/8 h (light/dark) photoperiod, light intensity (150 µmol/m^2^ s). A total of ten 2^nd^ instar larvae were added to the bottom, middle, and top trifoliate leaf surface for each plant. After 10 days of feeding, percent leaf defoliation was assessed for each plant. The percentage of leaf defoliation area was calculated in ImageJ software, as described (Ortega et al., 2016).

### Soybean cyst nematode (SCN; *H. glycines*) assays

Three independent transgenic soybean lines and six replicate plants for each line of individual TI gene construct under the control of CaMV 35S promoter were used for bioassays with SCN. The assay protocol was adapted according to Mazarei et al. (2011). Transgenic and wild-type soybean seeds were germinated in the potting mix for four days. Roots from each seedling were rinsed with water gently to remove potting mix and transferred to the pot containing sand: clay (in 3:1 ratio) mixture. One day after repotting, each seedling was inoculated with SCN HG type 0 (race 3). One hole, 1 cm deep, was made closure to the roots of seedlings. Each seedling was inoculated with 1 mL of inoculum with SCN eggs (approximately 2500). Plants were maintained in the growth chamber at 25 °C on a 16/8 h (light/dark) photoperiod with 140 µmol/m^2^ s light intensity. After five weeks, plant roots were individually washed with a strong jet of water to dislodge the cysts and females. These were counted under a stereomicroscope. The female index was calculated for each soybean line where the average number of mature female nematodes and cysts on the transgenic soybean line was divided by the average number of mature female nematodes and cysts on the susceptible soybean control line and multiplied by 100 (Lin et al., 2016).

### Growth characteristics of transgenic soybean plants

The effect of TI gene overexpression under the control of CaMV 35S promoter or rbcS-SRS4 promoter on soybean plant growth and development was evaluated under greenhouse conditions. The environmental conditions of the greenhouse were 16/8 h (light/dark) photoperiod and 25 °C temperature with fluctuations from a minimum of 22 °C to a maximum of 28 °C. Based on the results from insect feeding bioassays, plants overexpressing the two best-performing TI genes (KTI7 and BBI5) for each promoter type were chosen for growth and development studies with no herbivore application. Three independent T_3_ homozygous transgenic lines for each gene type were used in the experiment. A total of 40 plants were grown for each gene type (ten plants per transgenic and ten per non-transgenic control lines) to compare plants growth and development. Plants were started from seed in the greenhouse and grown until the seed was set. Several growth parameters including plant height, pods number, dry aboveground biomass yield, and total seed weight were analyzed.

### Corn earworm digestive enzyme inhibition assay

Midgut fluids from *H. zea* larvae (fourth instar) were used to assay trypsin inhibitory activity of transgenic plant extracts using the synthetic substrate BApNA. Midgut fluids were extracted from actively feeding 4th instar larvae. Ten larvae were chilled on ice and the guts containing the food bolus were carefully dissected and placed into a microcentrifuge tube. Immediately after collection of the 10 gut contents, the collection tube was submerged in liquid nitrogen and stored at -80 °C until used. Prior to assays, the gut content samples were thawed on ice and 500 μl of deionized water was added to each tube, vortexed for 2 min and centrifuged at 15,000 rpm for 20 min at 4 °C. The supernatants were filtered through 0.2 μm filters into new microcentrifuge tubes and considered as soluble proteinase samples. Protein concentrations were determined using the Qubit protein kit (Invitrogen) and adjusted to be 1 mg/ml using deionized water. The method of digestive enzyme inhibition analyses was conducted by following a previously described method. Total protein from six-week-old T3 transgenic and non-transgenic soybean plants was used for each gene construct. Three independent transgenic lines were used for each gene construct and six replicate plants were used for each line. All assays were carried out in triplicates from each plant.

### RNA extraction and expression analysis by qRT-PCR

TRI reagent (Zymo Research) was used to extract total RNA from six-week-old transgenic Arabidopsis and soybean plants according to the manufacturer’s protocol. Total RNA was quantified using a NanoDrop™ spectrophotometer ND-1000 (Thermo Fisher Scientific) and RNA integrity was checked by agarose gel electrophoresis. Total RNA was treated with DNaseI, and column purified with the RNA Clean & Concentrator™ kit (Zymo Research) to remove genomic DNA contamination. Total RNA (1 µg) was used to synthesize first-strand cDNA in a 20 µL reaction volume containing 1 µL of 50 µM oligo dT primer and 1 µL of 10 mM dNTP mix, 2 µL of 10×RT buffer, 4 µL of 25 mM MgCl2, 2 µL of 0.1 M DTT, 1 µL of RNaseOUT™ (40 U/µL), and 1 µL of SuperScript^®^ III RT (200 U/µL). The TI gene-specific primers (**Supplementary Table S1**) were designed for qRT-PCR using Primer3 (v 0.4.0). Real-time PCR was conducted in a 15 µL reaction volume containing, 7.5 µL of Power SYBR Green 2X Master Mix (Applied Biosystems, Foster City, CA, USA), 1 µL of cDNA (12.5 ng), 0.375 µL of each primer (10 µM), and 5.75 µL of H_2_O. The real-time PCR was carried out on a QuantStudio™ 6 Flex Real-Time PCR System (Applied Biosystems). Expression analyses were performed using a standard curve method for relative expression normalized to the Arabidopsis actin gene (*ACT*) for Arabidopsis and soybean ubiquitin gene (*GmUBI3*) for soybean plants.

## Results

### Endogenous expression patterns of trypsin inhibitor (TI) genes in non-transgenic soybean plants

**Figure 1:**
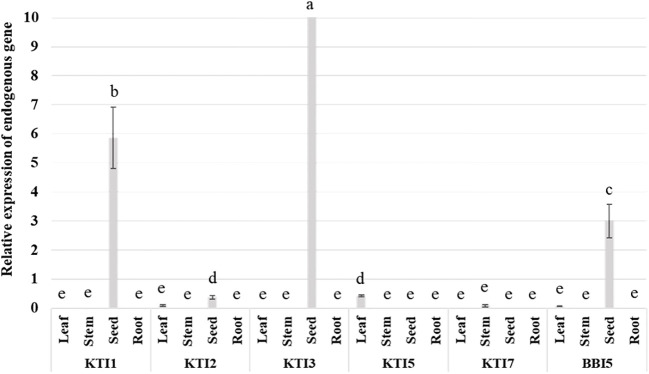
Endogenous expression patterns of the individual trypsin inhibitor genes (KTI1, KTI2, KTI3, KTI5, KTI7, BBI5) in different plant tissues of leaves, stems, and roots (six-week-old and seeds (30 days after flowering) wild-type soybean plants. The relative levels of transcripts were normalized to soybean ubiquitin gene (*GmUBI3*). Bars represent mean values of six biological replicates (plants) ± standard error. Bars with different letters are significantly different at *p* < 0.05 as tested by one-way analysis of variance followed by a Fisher’s least significant difference.

Quantitative reverse transcription-polymerase chain reaction (qRT-PCR) assays were used to determine the levels of expression for TI genes in leaves, stems, seeds, and roots of non-transgenic wild-type soybean plants. The expression of KTI1, KTI3, and BBI5 were the highest in seeds, whereas the expression of KTI2 was very low and KTI5, and KTI7 were undetectable in seeds (**Figure 1**). The expression of KTI1 and KTI3 were undetectable in leaves, stems, and roots. The expression of KTI5 was low and detected only in leaves. Similarly, the expression of KTI7 was low and detected only in stems (**Figure 1**). The expression of KTI2 and BBI5 were low and detected in leaves (**Figure 1**). There was no detectable expression for any of the TI genes in roots (**Figure 1**).

### Transient expression analysis of trypsin inhibitor (TI) genes in *Nicotiana benthamiana*

Agroinfiltrated *N. benthamiana* leaves containing the individual 35S::TI gene construct were evaluated for transient expression. The relative qRT-PCR expression levels for KTI2, KTI3, and KTI7 were the highest (3.75, 3.86, and 3.35, respectively), and for KTI1, KTI5, and BBI5 were low (0.21, 0.12, and 0.73, respectively) (**Supplemental Figure S5**).

### Overexpression of trypsin inhibitor (TI) genes in transgenic Arabidopsis and soybean plants

Six independent T_1_ transgenic Arabidopsis and soybean lines were generated for each TI gene construct under the control of either 35S or rbcS-SRS4 promoters. Subsequently, five independent homozygous T_3_ transgenic Arabidopsis and soybean lines were obtained and screened by PCR using genomic DNA to confirm the presence of transgenes *Bar* and TI (**Supplemental Figures S2–4**). For further analysis, three independent homozygous T_3_ transgenic Arabidopsis and soybean lines were selected to represent a range of transgene expression for each TI gene.

### Gene expression analysis in transgenic Arabidopsis overexpressing trypsin inhibitor (TI) genes

The relative qRT-PCR transcript abundance of each TI transgene (under the control of 35S promoter) was determined in three independent homozygous T_3_ transgenic Arabidopsis lines. Considerable expression levels of KTI1, KTI2, KTI3, KTI5, KTI7, and BBI5 were observed in leaves of all three corresponding transgenic lines (**Supplemental Figure S6**). In particular, the relative expression of KTI7 was found to be the highest (397.72 ± 63.56) among TI genes (**Supplemental Figure S6E**). The high expression levels of KTI7 were consistent among three transgenic lines. The expression of KTI3 and BBI5 were medium (15.35 ± 2.65 and 13.03 ± 2.69, respectively) (**Supplemental Figures S6C–F**), whereas the expression of KTI1, KTI2, and KTI5 were low (3.87 ± 0.56, 6.82 ± 2.36, and 1.72 ± 0.49, respectively) (**Supplemental Figures S6A, B-D**). No expression was detected in the non-transgenic (WT) plants (**Supplemental Figure S6**).

### Gene expression analysis in transgenic soybean overexpressing trypsin inhibitor (TI) genes

**Figure 2:**
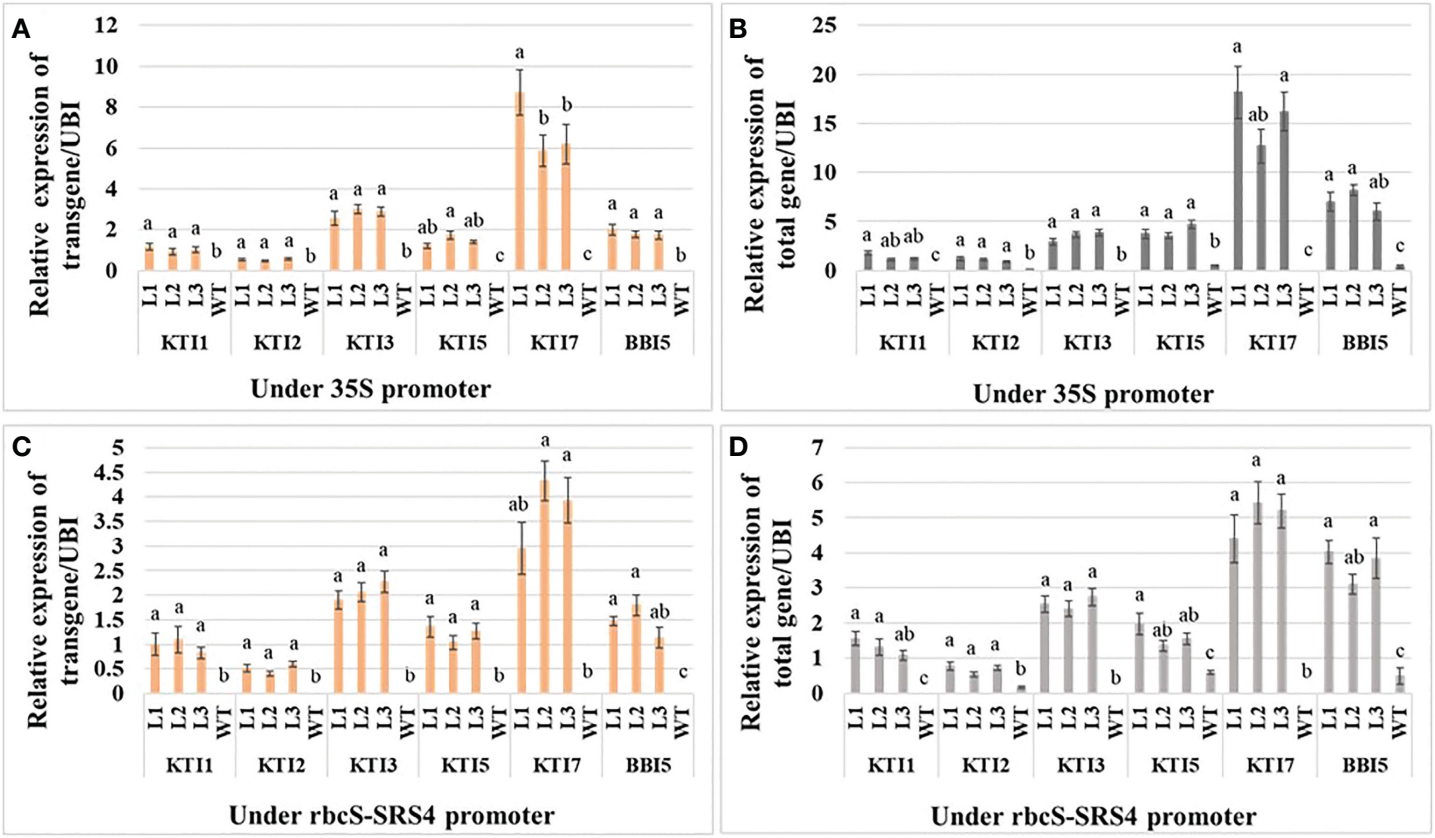
Expression analysis of the individual trypsin inhibitor gene construct in leaves of six-week-old T_3_ transgenic and non-transgenic wild-type (WT) soybean plants. The relative expression of transgene **(A)** and total gene **(B)** corresponding to KTI1, KTI2, KTI3, KTI5, KTI7, and BBI5 under the control of 35S CaMV promoter. The relative expression of transgene **(C)** and total gene **(D)** corresponding to KTI1, KTI2, KTI3, KTI5, KTI7, and BBI5 under the control of rbcS-SRS4 promoter. The relative levels of transcripts were normalized to soybean ubiquitin gene (*GmUBI3*). Bars represent mean values of six biological replicates (plants) per each independent line (L1, L2, L3) ± standard error. Each TI gene was statistically analyzed separately. Bars with different letters are significantly different at *p* < 0.05 as tested by one-way analysis of variance followed by a Fisher’s least significant difference.

The relative qRT-PCR expression of each TI transgene and total gene (under the control of the 35S promoter) was determined in three independent homozygous T_3_ transgenic soybean lines. Similar to transgenic Arabidopsis, the relative expression of the transgene and total gene for KTI7 was the highest (transgene: 6.92 ± 0.96 and total gene: 15.68 ± 2.11) in soybean among TI genes (**Figures 2A, B**). The relative expression of the transgene and total gene for KTI3, KTI5, and BBI5 were medium with 2.4-, 4.80-, and 3.70-fold, respectively, lower transgene expression and 4.6-, 3.9-, and 2.2-fold, respectively, lower total gene expression compared to KTI7 (**Figures 2A, B**). The expressions of KTI1 and KTI2 were low (transgene: 6.6- and 13-fold lower compared to KTI7) (total gene: 11- and 14-fold lower compared to KTI7) in transgenic soybean lines (**Figures 2A, B**). The relative expression in all transgenic lines was significantly different compared to non-transgenic (WT) plants (**Figures 2A, B**). Similarly, the relative expression of each TI transgene and total gene (under the control of the rbcS-SRS4 promoter) had similar patterns in transgenic soybean lines (**Figures 2C, D**). The expression of the transgene and total gene for KTI7 was the highest (transgene: 3.74 ± 0.46 and total gene: 5.00 ± 0.59) in soybean lines among TI genes (**Figures 2C, D**). The expression of the transgene and total gene for KTI3, KTI5, and BBI5 were medium with 1.8-, 3-, and 2.5-fold, respectively, lower transgene expression and 2-, 3-, and 1.4-fold, respectively, lower total gene expression compared to KTI7 (**Figures 2C, D**). Whereas the expression of KTI1 and KTI2 were low (transgene: 3.8- and 7.5-fold lower compared to KTI7) (total gene: 3.9- and 8-fold lower compared to KTI7) in transgenic soybean lines (**Figures 2C, D**). The relative expression in all transgenic lines was significantly different compared to non-transgenic (WT) plants (**Figures 2C, D**).

### Enzyme inhibitory activity

**Figure 3:**
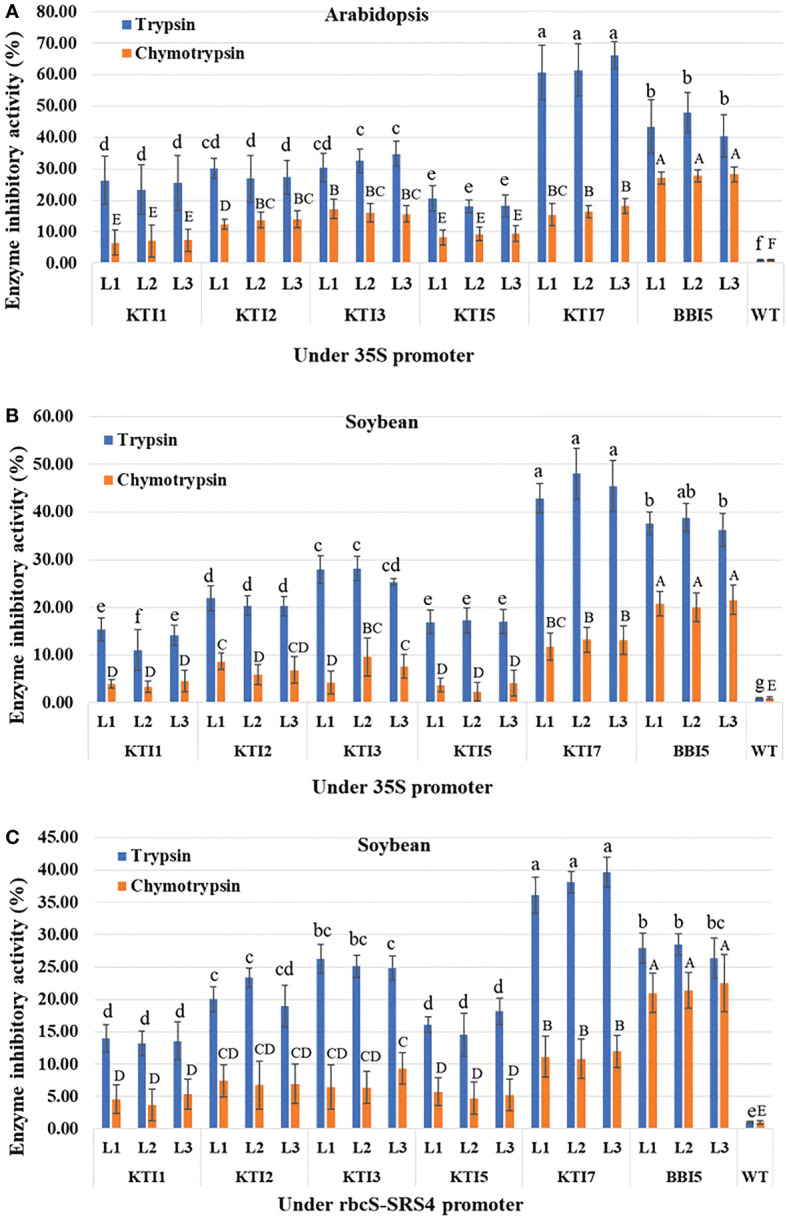
Enzyme inhibitory activity of the individual trypsin inhibitor gene (KTI1, KTI2, KTI3, KTI5, KTI7, BBI5) construct in leaves of six-week-old T_3_ transgenic **(A)** Arabidopsis plants under the control of 35S CaMV promoter, **(B)** Soybean plants under the control of 35S CaMV promoter, and **(C)** Soybean plants under the control of rbcS-SRS4 promoter. Percentage of trypsin and chymotrypsin inhibition activities in leaf total protein extract from transgenic plants with corresponding each type of gene relative to that of non-transgenic wild-type plants. Bars represent mean values of six biological replicates (plants) per each independent line (L1, L2, L3) ± standard error. Bars with different letters are significantly different at *p* < 0.05 as tested by one-way analysis of variance followed by a Fisher’s least significant difference. Bars with lowercase letters represent trypsin group and uppercase letters represent chymotrypsin group.

*In vitro* enzyme inhibitory activity (trypsin and chymotrypsin) was determined using total protein from transgenic Arabidopsis and soybean lines overexpressing the individual TI gene construct. For transgenic Arabidopsis lines overexpressing the 35S::TI gene construct, transgenic lines of KTI7 (line 1: 60.75 ± 8.68%, line 2: 61.45 ± 8.36%, and line 3: 66.06 ± 4.31%) and BBI5 (line 1: 43.37 ± 8.53%, line 2: 47.96 ± 6.35%, and line 3: 40.45 ± 6.69%) showed higher inhibitory activity against commercial trypsin among all TI genes (**Figure 3A**). Transgenic Arabidopsis lines of KTI1 (an average of 2.5-fold), KTI2 (2.2-fold), and KTI3 (1.9-fold) showed moderate inhibitory activity, whereas KTI5 (3.3-fold) showed lower inhibitory activity (**Figure 3A**) compared to KTI7. Transgenic Arabidopsis lines of BBI5 showed higher inhibitory activity (27.73 ± 2.08%) against commercial chymotrypsin, whereas KTI2 (2-fold), KTI3 (1.7-fold), and KTI7 (1.6-fold) had moderately low activity compared to BBI5. The KTI1 (4-fold) and KTI5 (3-fold) had much lower inhibitory activity compared to BBI5 (**Figure 3A**).

For transgenic soybean lines overexpressing the 35S::TI gene construct, transgenic lines of KTI7 and BBI5 showed the highest trypsin inhibitory activity (an average of 45.44 ± 4.55% and 37.53 ± 2.92%, respectively) among TI genes (**Figure 3B**). Transgenic soybean lines of KTI1 (3.3-fold), KTI2 (2.2-fold), KTI3 (1.6-fold), and KTI5 (2.6-fold) showed lower trypsin inhibitory activity compared to KTI7 lines (**Figure 3B**). Transgenic soybean lines of BBI5 showed the highest chymotrypsin inhibitory activity (an average of 20.78 ± 2.85%), whereas KTI1, KTI2, KTI3, KTI5, and KTI7 showed an average of 7-, 3-, 2.9-, 6-, 1.6-fold respectively lower inhibitory activity compared to BBI5 (**Figure 3B**).

For transgenic soybean lines overexpressing the rbcS-SRS4::TI gene construct, transgenic lines of KTI7 showed the highest trypsin inhibitory activity (an average of 37.97 ± 2.23%) (**Figure 3C**). In contrast, transgenic soybean lines of KTI1, KTI2, KTI3, KTI5, and BBI5 showed an average of 2.8-, 1.8-, 1.5-, 2.3-, and 1.4-fold, respectively lower inhibitory activity compared to KTI7 (**Figure 3C**). The chymotrypsin inhibitory activity was found to be higher in transgenic soybean lines of BBI5 (an average of 21.63 ± 3.40%), whereas transgenic lines KTI1, KTI2, KTI3, KTI5, and KTI7 showed an average 4.7-, 3-, 2.9-,4-, 1.9-fold, respectively lower inhibitory activity compared to transgenic BBI5 lines (**Figure 3C**).

### Detached leaf punch feeding bioassay

Homozygous T_3_ transgenic Arabidopsis and soybean lines overexpressing the individual TI gene construct were used to determine the effect of corn earworm by feeding leaf punches. Leaf punches from transgenic Arabidopsis lines overexpressing the 35S::TI gene and non-transgenic Arabidopsis were fed by corn earworm first instar larvae (**Supplemental Figures S7A, B**). Upon eight days of feeding, the representative larval size was shown in **Supplemental Figure S7C**. Larval weight was recorded and compared between transgenic lines of each TI gene and non-transgenic plants. Notably, all transgenic lines of each TI gene showed a significant reduction of larval weight compared to non-transgenic (WT) plants (**Supplemental Figure S7**). Particularly, transgenic KTI7 and BBI5 lines had the greatest reduction in corn earworm larval biomass, with an average of 8.55 ± 1.20 mg and 10.09 ± 1.58 mg larval weight, respectively, compared to non-transgenic plants (an average of 23.63 ± 0.82 mg) (**Supplemental Figure S7D**). Transgenic Arabidopsis lines of KTI1, KTI2, KTI3, and KTI5 showed a significant reduction of larval weight, an average of 14.87 ± 1.07 mg, 11.72 ± 1.4 mg, 11.09 ± 1.31 mg, and 12.93 ± 1.49 mg respectively (**Supplemental Figure S7D**).

**Figure 4:**
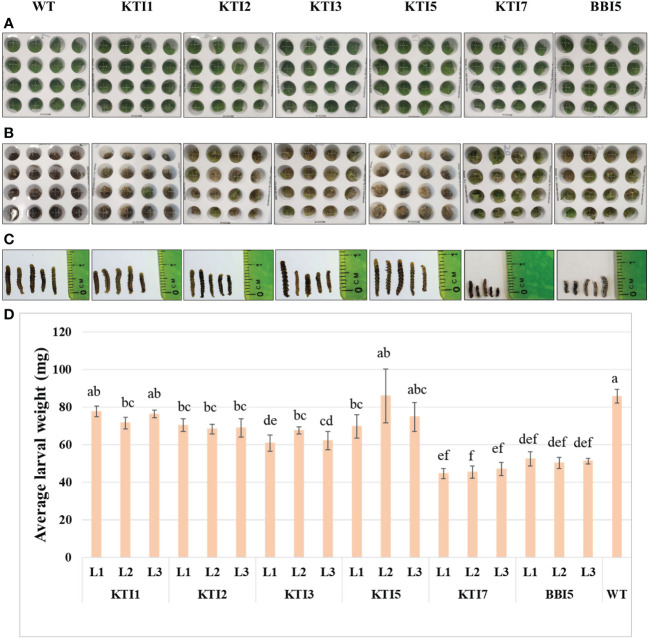
Detached leaf-punch bioassay in six-week-old T_3_ transgenic soybean (under the control of CaMV 35S promoter) using corn earworm (*Helicoverpa zea*) neonate larvae. **(A)** Wild-type and transgenic soybean plants detached-leaf punches before corn earworm larval inoculation. **(B)** Leaf punches were inoculated with corn earworm neonate larvae and eight days after feeding. **(C)** Larval size after eight days of feeding. **(D)** Average larval weight after eight days of feeding in WT and transgenic lines (L1, L2, and L3). Bars represent mean values of six biological replicates ± standard error. Bars with different letters are significantly different at *p* < 0.05 as tested by one-way analysis of variance followed by a Fisher’s least significant difference.

Transgenic soybean lines overexpressing the 35S::TI gene were also used for leaf punch feeding assays (**Figures 4A–C**). Transgenic soybean lines of KTI7 and BBI5 had a significant reduction of larval weight (45.80 ± 3.16 mg and 51.42 ± 2.73 mg, respectively) compared to non-transgenic control plants (85.88 ± 3.71 mg) (**Figure 4D**). Larvae fed on other transgenic soybean lines of KTI1, KTI2, KTI3, and KTI5 showed no consistent significant differences in the larval weight compared to non-transgenic control plants (**Figure 4D**).

**Figure 5:**
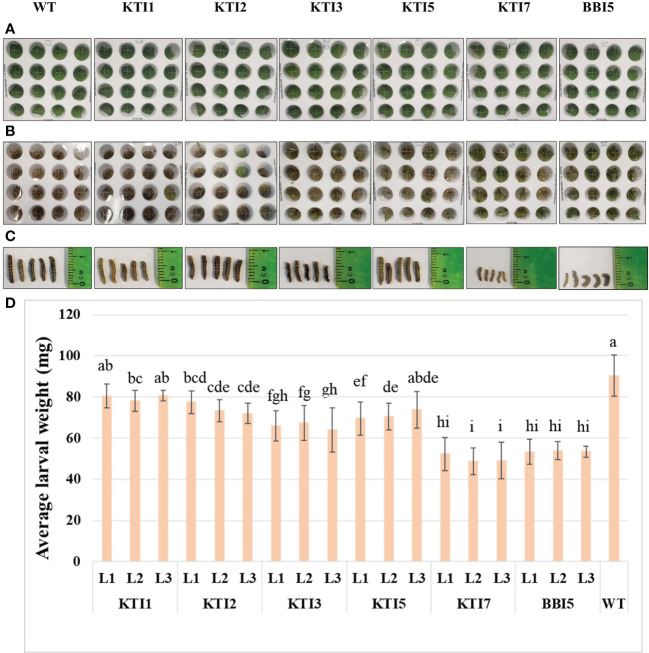
Detached leaf-punch bioassay in six-week-old T_3_ transgenic soybean (under the control of rbcS-SRS4 promoter) using corn earworm (*Helicoverpa zea*) neonate larvae. **(A)** Detached leaf-punches from wild-type and transgenic plants. **(B)** Neonate corn earworm larvae were inoculated in each well and showing after eight days feeding. **(C)** Representative corn earworm larval size after eight days of feeding. **(D)** Average larval weight after feeding in WT and transgenic lines (L1, L2, and L3). Bars represent mean values of six biological replicates ± standard error. Bars with different letters are significantly different at *p* < 0.05 as tested by one-way analysis of variance followed by a Fisher’s least significant difference.

The leaf punch feeding assays were also performed on transgenic soybean lines overexpressing the rbcS-SRS4::TI gene (**Figures 5A–C**). Similarly, transgenic soybean lines of KTI7 and BBI5 reduced larval weight (50.00 ± 7.76 mg and 53.54 ± 4.32 mg, respectively) compared to non-transgenic control plants (90.3 ± 10.06 mg) (**Figure 5D**). There were no consistent significant differences for other transgenic soybean lines of KTI1, KTI2, KTI3, and KTI5 (**Figure 5D**). Based on these results, two best performing types of transgenic plants overexpressing KTI7 and BBI5 genes under the control of either 35S or rbcS-SRS4 promoter were selected for further evaluation.

### Whole plant feeding soybean bioassays for corn earworm

**Figure 6:**
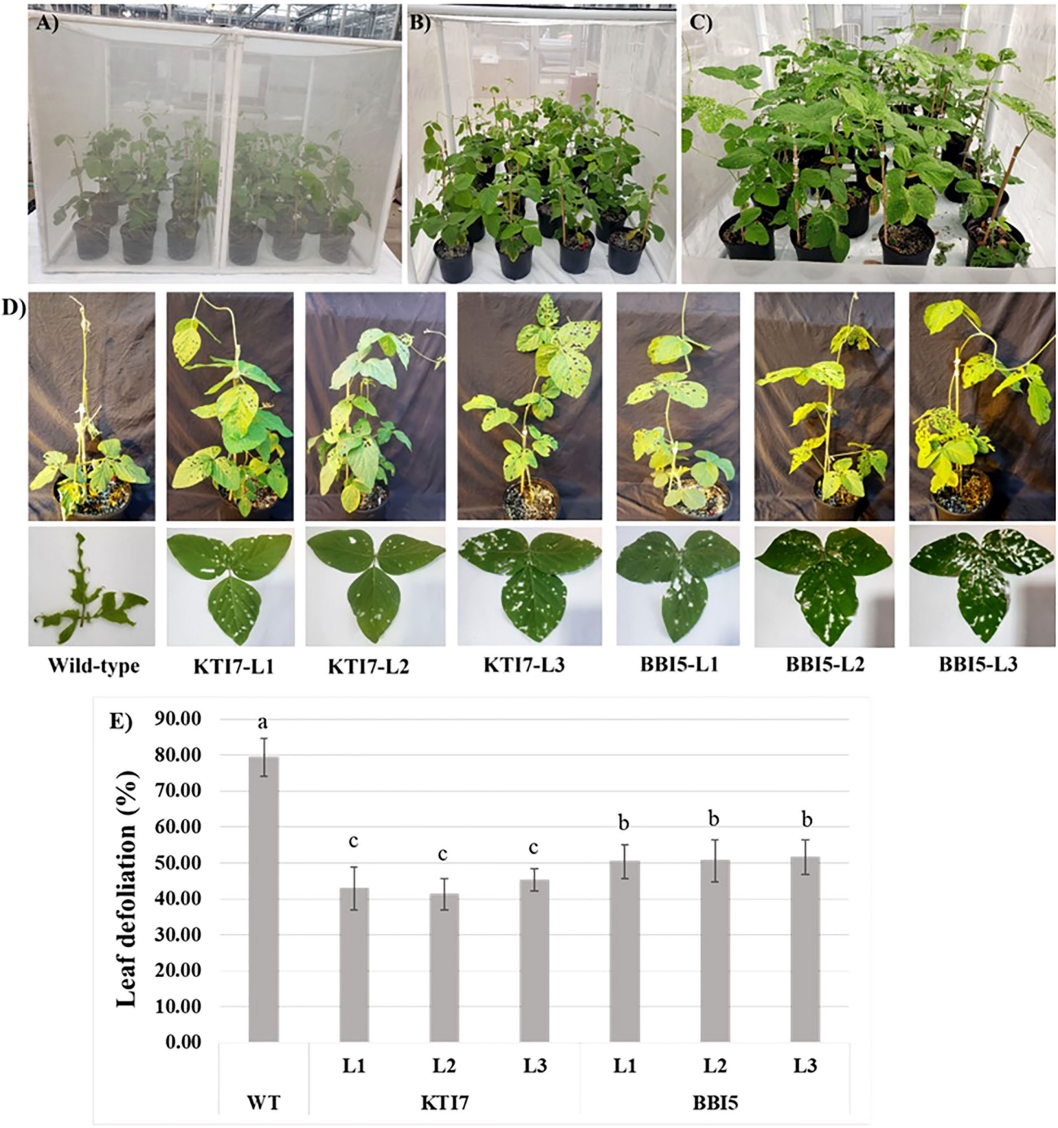
Whole plant feeding bioassay using six-week-old T_3_ transgenic soybean plants that contain the 35S cauliflower mosaic virus promoter driving the expression of individual trypsin inhibitor genes (KTI7 and BBI5) grown under greenhouse conditions with corn earworm (*Helicoverpa zea*) second instar larvae. **(A)** Representative of transgenic and non-transgenic wild-type soybean plants in a polyester-mesh cage. **(B)** Ten larvae were added to each plant. **(C)** Ten days after larvae feeding. **(D)** Representative defoliated wild-type and transgenic plants. **(E)** Leaf defoliation rate after 10 days of larvae feeding of wild-type (WT) and transgenic lines. Bars represent mean values of six biological replicates (plants) per each independent line (L1, L2, L3) ± standard error. Bars with different letters are significantly different at *p* < 0.05 as tested by one-way analysis of variance followed by a Fisher’s least significant difference.

**Figure 7:**
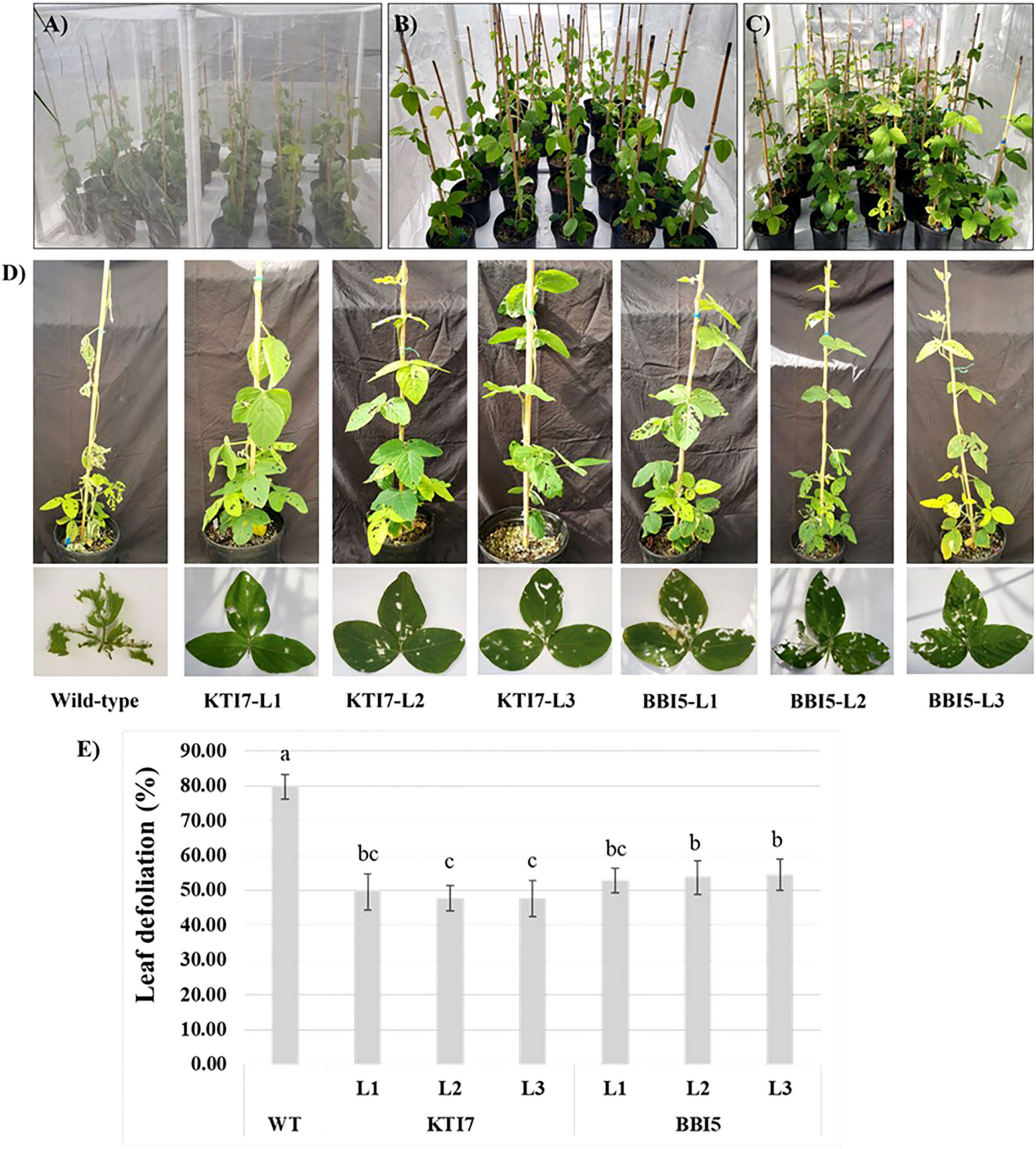
Whole plant feeding bioassay using six-week-old T_3_ transgenic soybean plants that contain the rbcS-SRS4 promoter driving the expression of trypsin inhibitor genes (KTI7 and BBI5) grown under greenhouse conditions with corn earworm (*Helicoverpa zea*) second instar larvae. **(A)** Representative of transgenic and non-transgenic wild-type soybean plants in a polyester-mesh cage. **(B)** Ten larvae were added to each plant. **(C)** Ten days after larvae feeding. **(D)** Representative defoliated wild-type and transgenic plants. **(E)** Leaf defoliation rate after 10 days of larvae feeding of wild-type (WT) and transgenic lines. Bars represent mean values of six biological replicates (plants) per each independent line (L1, L2, L3) ± standard error. Bars with different letters are significantly different at *p* < 0.05 as tested by one-way analysis of variance followed by a Fisher’s least significant difference.

The corn earworm bioassays were performed on plants grown in a polyester-mesh cage under greenhouse conditions (**Figure 6**). Homozygous T_3_ transgenic soybean lines for KTI7 and BBI5 (under the control of 35S promoter) and non-transgenic control plants were inoculated with corn earworm second instar larvae (**Figures 6A, B**). After ten days of feeding, the percent leaf defoliation was assessed for each plant (**Figure 6C**). Representative defoliated transgenic, and non-transgenic plants are shown in **Figure 6D**. Both transgenic KTI7 and BBI5 lines showed a significant reduction in leaf defoliation (KTI7: line 1, 42.89 ± 6.00%; line 2, 41.20 ± 4.4%; line 3, 45.29 ± 3.07%) (BBI5: line 1, 50.37 ± 4.65%; line 2, 50.60 ± 5.83%; and line 3, 51.64 ± 4.84%), compared to non-transgenic control plants (79.53 ± 5.29%) (**Figure 6E**). Transgenic soybean lines for both KTI7 and BBI5 driving under the control of the rbcS-SRS4 promoter and non-transgenic control plants were also used for corn earworm feeding bioassay (**Figures 7A–C**). Representative defoliated plants for transgenic KTI7 and BBI5 lines, and non-transgenic control plants are shown in **Figure 7D**. Similarly, both KTI7 and BBI5 transgenic lines showed a significant reduction in leaf defoliation for KTI7 lines (line 1: 49.51 ± 5.10%, line 2: 47.66 ± 3.64%, and line 3: 47.60 ± 5.10%) and BBI5 lines (line 1: 52.79 ± 3.57%, line 2: 53.69 ± 4.78%, and line 3: 54.51 ± 4.48%), compared to non-transgenic control plants (79.74 ± 5.48%) (**Figure 7E**).

### Effect of soybean cyst nematode (SCN) bioassay

Homozygous T_3_ transgenic soybean lines for KTI7 and BBI5 (under the control of 35S promoter) were used for SCN HG Type 0 (race 3) bioassay. Both transgenic KTI7 and BBI5 lines showed no significant differences in the female index compared to non-transgenic control plants (**Supplemental Figure S8**).

### Agronomic trait evaluation under greenhouse conditions

Homozygous T_3_ transgenic soybean lines for KTI7 and BBI5 (under the control of either 35S or rbcS-SRS4 promoter) and non-transgenic control plants were grown under greenhouse conditions to evaluate agronomic traits for plant height, number of pods, dry aboveground biomass, and seed yield per plant. The representative transgenic plants under the control of two different promoters were shown in **Supplemental Figures S9A, B**. There were no significant differences in the plant height, pod numbers, dry aboveground biomass, and seed yield per plant comparing transgenic and non-transgenic plants (**Supplemental Figures S9C–F**). Furthermore, there was no variation in agronomic traits among transgenic lines (**Supplemental Figures S9C–F**). There was also no negative effect on plant growth and development compared to non-transgenic plants.

### Inhibition of gut enzymes

The effect of trypsin inhibitor genes on the inhibition of gut enzymes has been performed. The results showed the highest inhibition of gut enzymes by KTI7 (line 1: 28.95 ± 2.65, line 2: 32.15 ± 2.64, and line 3: 29.03 ± 3.03) and BBI5 (line 1: 20.84 ± 1.52, line 2: 20.61 ± 2.08, and line 3: 19.67 ± 0.92) compared to non-transgenic control (1.00± 0.01) (**Supplemental Figure S10**). The proteolytic inhibition by KTI1, KTI2, KTI3, and KTI5 showed an average of 4-, 3-, 3-, 7- fold, respectively lower compared to KTI7 (**Supplemental Figure S10**).

## Discussion

Soybean trypsin inhibitors (TIs) are considered antinutritional factor on herbivore metabolism and development. The expression of TIs in soybean seed and its potential as anti-herbivory strategy in different host plants have been well studied (Larocque and Houseman, 1990; Lee et al., 1999; Marchetti et al., 2000; Falco and Silva-Filho, 2003; Franco et al., 2004; Vasudev and Sohal, 2016). However, the low quantities of TIs naturally found in soybean leaves and stems limit their endogenous effectiveness in deterring leaf defoliating insects. The present study demonstrates the potential of increasing the expression of TIs soybean to higher levels in shoots to increase plant defense against insects in soybean.

With a view to study the TI genes in soybean, in addition to the three known soybean trypsin inhibitors KTI1, KTI2 and KTI3 (Lee et al., 1999; Marchetti et al., 2000; Falco and Silva-Filho, 2003), we identified novel KTI5, KTI7, and BBI5 genes. Transcript expression analysis revealed that expression of KTI1, KTI3, and BBI5 were highest in seeds. Yet, expression of KTI1, KTI2 and KTI3, KTI5, KTI7, and BBI5 were low or undetectable in leaves and stems. No detectable expression for any of the TI genes was observed in roots (**Figure 1**). These results indicate there is low expression of native TI genes in shoot tissues, limiting their involvement in defense against leaf defoliating insects.

Varied expression levels of the individual TI genes driven by 35S promoter were observed in transiently transformed *N. benthamiana* leaves. The expression of KTI2, KTI3, and KTI7 were the highest, whereas for KTI1, KTI5, and BBI5 were lower (**Supplemental Figure S5**). The varied expression profile driven by the 35S promoter in different tissue types and plant species was also shown in other studies (Benfey and Chua, 1989; Sunilkumar et al., 2002; Kiselev et al., 2021).

We also observed varied expression patterns of the individual TI genes driven by the constitutive 35S or green-tissue rbcS-SRS4 promoter in leaves of stable transgenic Arabidopsis or soybean lines. Yet, the high expression levels were consistently observed in leaves of KTI7 and BBI5 overexpressing lines, regardless of the plant species or promoter type (**Figure 2**; **Supplemental Figure S6**). We also found a positive association between the expression level of TI genes and trypsin and chymotrypsin enzyme inhibitory activities in transgenic plants as higher expressor lines also had the higher enzyme inhibitory activity in leaves (**Figures 2**, **3**). These results demonstrate that the overexpression of the TI genes has led to the production of functional inhibitory enzymes in leaves of transgenic plants as a possible defense strategy against defoliating insects. The functional enzyme activity is of most interest as low inhibitor expression levels in transgenic plants were shown to have low to no inhibitory activity against digestive enzymes (De Leo et al., 1998; De Leo et al., 2001; Alvarez-Alfageme et al., 2007).

Detached leaf punch insect bioassays resulted in a variable degree of larval weight reduction in transgenic Arabidopsis TI-overexpressing lines (**Supplemental Figure S7**). A negative association between enzyme inhibitory activity and larval weight was observed in the transgenic lines. For example, herbivores feeding on transgenic KTI7 and BBI5 lines with the highest enzyme inhibition activity also had the lowest larval weight (**Figure 3A**; **Supplemental Figure S7**). This negative association between enzyme inhibitory activity and larval weight was also observed for KTI1, KTI2, KTI3, and KTI5 overexpressing lines (**Figure 3A**; **Supplemental Figure S7**). These results suggest that the TI genes are possibly involved in conferring defense against defoliating insects. Consistently, previous studies have shown the role of KTI1, KTI2, and KTI3 in defense response against several insects on different host plants (Lee et al., 1999; Marchetti et al., 2000; Falco and Silva-Filho, 2003). The BBI gene was also shown to play a role in defense against insects (Bowman, 1946; Ye et al., 2001; Azzouz et al., 2005; Kaur et al., 2017; Lokya et al., 2020).

Detached leaf punch insect bioassays of transgenic soybean overexpressing TI genes also resulted in a variable degree of larval weight reduction under control of either the constitutive 35S or green-tissue rbcS-SRS4 promoter (**Figures 4**, **5**). Similar to Arabidopsis, larvae feeding on transgenic KTI7 and BBI5 soybean lines had the highest reduction of larval weight as compared with those fed on non-transgenic leaves (**Figures 4**, **5**). No obvious effect on larval weight was observed on the transgenic KTI1, KTI2, KTI3, and KTI5 (**Figures 4**, **5**). Previous studies have also shown that the larval weight was decreased to the increased level of transgene expression in plant (Gatehouse et al., 1994; De Leo et al., 1998; Marchetti et al., 2000; Brunelle et al., 2004; Abdeen et al., 2005).

We also observed dissimilarity in TI gene identity effects on larval weight between transgenic soybean and Arabidopsis plants. This dissimilarity perhaps is related to expression levels and enzyme inhibitory activity. Transgenic Arabidopsis conferred higher transgene expression and higher enzyme inhibitory activity for individual TI genes compared to transgenic soybean plants. Nevertheless, our results indicate that overexpression of KTI7 and BBI5 consistently led to reduced larval growth in both plant species, which further points out to the potential application of these soybean TI genes in other plant species. Furthermore, our results provide additional knowledge to the biotechnological applications of defensive chemicals as an effective anti-herbivory strategy in plants (Lee et al., 1999; Franco et al., 2004; Quilis et al., 2014; Ni et al., 2017; Zhou et al., 2017; Ibrahim et al., 2020).

The top performing transgenic soybean KTI7 and BBI5 overexpressing lines under control of either constitutive 35S or green-tissue rbcS-SRS4 promoter were used for whole plant feeding insect bioassays under greenhouse conditions. Remarkably, the results demonstrated a significant reduction of leaf defoliation (35S-KTI7 = 43.12 ± 4.49%, 35S-BBI5 = 50.87 ± 5.11%, rbcS-SRS4-KTI7 = 48.26 ± 4.61%, and rbcS-SRS4-BBI5 = 53.67 ± 4.28%) compared to non-transgenic plants (80.84 ± 3.48%) (**Figures 6**, **7**). Although several *in vitro* studies have shown the overexpression of TI gene for the improvement of defense against insects (Azzouz et al., 2005; Srinivasan et al., 2005; Kaur et al., 2017; Golla et al., 2018), the evaluation on whole plant bioassay under greenhouse or field conditions is limited (Qiu, 2008). Our findings further confirm the effectiveness of these soybean TI genes in greenhouse infestation of plants with insects. We observed no significant differences in leaf defoliation between 35S or rbcS-SRS4 promoter driven TI genes (**Figures 6**, **7**). Our results showed that green tissue-specific promoter rbcS-SRS4 achieved a similar effect of leaf defoliation. Green tissue specific promoters direct the expression preferentially in shoots and limit the expression in roots, where TI production is not needed. A previous study also showed similar gene expression patterns in leaves comparing 35S and rbcS-SRS4 promoter (Cui et al., 2015).

Our results involving transgenic soybean KTI7 and BBI5 plants and SCN bioassay showed no effect of gene overexpression on SCN infection as there were no differences in SCN female index between transgenic and non-transgenic plants (**Supplemental Figure S8**). Even though the reduced nematode infestations using trypsin inhibitors has been reported (Atkinson et al., 1995), one possible explanation can be that the expression levels of KTI7 and BBI5 in the transgenic roots were not sufficient to confer the SCN defense (**Supplemental Figure S11**). Yet, the role of TI genes in plant-nematode interaction remains unknown and further study of these genes would provide insights into the interactions between plants and nematodes.

Of additional interest, several agronomic traits were evaluated in KTI7 and BBI5 overexpressing transgenic soybean lines. The phenotypes and vegetative growth of transgenic KTI7 and BBI5 soybean lines were similar to that of the non-transgenic plants in the absence of herbivory. No adverse effects of TI gene overexpression were observed on plant height, pods number, seed weight, and dry aboveground biomass yield (**Supplemental Figure S9**). Previous studies also showed no negative effects of transgene expression on growth and development of plant (Brunelle et al., 2004; Rivard et al., 2006; Badri et al., 2009; Quain et al., 2014).

In addition to the effects of gut enzyme inhibition, this study demonstrated the sensitivity of TI genes to gut extracts. Overall, the KTI7 and BBI5 showed the highest trypsin inhibitory activity compared to non-transgenic control. These findings support the results of using commercial trypsin in response to TI genes. These results indicated that the KTI7 and BBI5 interfere with the digestive mechanism by inhibiting enzyme activity.

In conclusion, we have demonstrated that the KTI7 and BBI5 genes encoding TI inactivate insect digestive enzymes *via* both *in vitro* enzyme assays and insect bioassays using transgenic Arabidopsis and soybean lines. The findings in the present study could be potentially used to enhance resistance against leaf defoliating insects. The utilization of inherent defensive proteins in plants has potential benefit to agricultural crop protection and environment with reduced chemical application.

## Data availability statement

The raw data supporting the conclusions of this article will be made available by the authors, without undue reservation.

## Author contributions

MS designed and performed experiments, analyzed data, produced figures, and wrote the manuscript. MM participated in the data analysis and interpretation and assisted with writing and revising the manuscript. JJ-F provided insect eggs and assisted in conducting insect experiments, participated in data interpretation, and assisted with revising the manuscript.TH assisted in conducting nematode experiments and interpreted the results. RM participated in the experimental design, assisted with data analysis, and assisted with writing and revising the manuscript. CNS conceived and designed the study, acquired funding, assisted with the interpretation of results, and helped in editing and revising the manuscript. All authors contributed to the article and approved the final version.
