# Supplementary material for: Overexpression of soybean trypsin inhibitor genes decreases defoliation by corn earworm (Helicoverpa zea) in soybean (Glycine max) and Arabidopsis thaliana
Source: Front Plant Sci. 2023 Feb 17;14:1129454. doi: 10.3389/fpls.2023.1129454 (PMC9982021; doi:10.3389/fpls.2023.1129454)
Supplement: Supplementary file 1 [file DataSheet_1.docx]

**Supplementary Table (S1):** **List of primers used in vector construction**

Restriction enzymes sites are underlined.

| **Cloning gene/promoter** | **Primer** |
| --- | --- |
| KTI1 | F- CACCATACTAGTTACTATCTTCTTTGCTCTCTTTCTAGT |
|  | R- CACTACGGCGCGCCTGATGATCTAAATTTCTGAAACTGA |
| KTI2 | F- CACCTCACTAGTATGAAGCCTACCCTACTATTATCCCTTTCC |
|  | R- CACTACGGCGCGCCTCAAACTACGGACTTGATAATTCCATCAACC |
| KTI3 | F- CACCTCACTAGTATGAAGAGCACCATCTTCTTTGCTCT |
|  | R- CACTACGGCGCGCCTCACTCACTGCGAGAAAGGC |
| KTI5 | F- CACCTACTAGTATGAAGAGCACTACCTTGTTCGCT |
|  | R- CACTACGGCGCGCCTCACTCACTGGCAGGAAGCAC |
| KTI7 | F- CACCTACTAGTATGAAGAATACTATCTTCTTCGCTCTCTTTCTTG |
|  | R- CACTACGGCGCGCCTCAAGCAGTAGCTGATGATTCAACTT |
| BBI5 | F- CACCATACTAGTAGCTCTACCTTGTTCGCT |
|  | R- CACTACGGCGCGCCCGCCAGGAAGCAA |
| rbcS-SRS4 | F- CACCGAGCTCGTGGATGACTCAAGTGCTGG |
|  | R- CACTACTAGTTGCATTGCACTCTTCCACCG |

**Supplementary Table (S2):** **Lists of primers used in *Nicotiana benthamiana* and Arabidopsis PCR and qRT-PCR**

| **Gene** | **Forward primer** | **Reverse primer** |
| --- | --- | --- |
| KTI1 | TTTGTGCCTTCACCATCTCA | GGCGATAAAAAGGGCATGTA |
| KTI2 | CCTTTCCTTCCTGCCTCTCT | GGCCTAGTTTCAATCCACCA |
| KTI3 | CTTTTGTGCCTTCACCACCT | GATTCGATATGGGGACGAGA |
| KTI5 | TTACAAGAGGAAGCGGTGGT | CAATAGCCCACCAAAGAGGA |
| KTI7 | AGAGGAGACGGAGGTGGAAT | GGCACAAAAGTGAACCCAAT |
| BBI5 | CCATCCTTTGAGCATTTCGT | TGTGCTACCCTCAAGCAGTG |
| GAPDH | AGCTCAAGGGAATTCTCGATG | AACCTTAACCATGTCATCTCCC |
| ACT | ACCTTGCTGGACGGACCTTACTGAT | GTTGTCTCGTGGATTCCAGCAGCTT |

**Supplementary Table (S3):** **List of primers used in soybean PCR and qRT-PCR**

| **Gene** | **Forward primer** | **Reverse primer** |
| --- | --- | --- |
| Endo-KTI1 | TTTGTGCCTTCACCATCTCA | GGCGATAAAAAGGGCATGTA |
| Endo-KTI2 | CCTTTCCTTCCTGCCTCTCT | GGCCTAGTTTCAATCCACCA |
| Endo-KTI3 | CTTTTGTGCCTTCACCACCT | GATTCGATATGGGGACGAGA |
| Endo-KTI5 | TTACAAGAGGAAGCGGTGGT | CAATAGCCCACCAAAGAGGA |
| Endo-KTI7 | AGAGGAGACGGAGGTGGAAT | GGCACAAAAGTGAACCCAAT |
| Endo-BBI5 | CCATCCTTTGAGCATTTCGT | TGTGCTACCCTCAAGCAGTG |
| Bar | GAAGTCCAGCTGCCAGAAAC | AAGCACGGTCAACTTCCGTA |
| chvA | CGAAACGCTGTTCGGCCTGTGG | GTTCAGCAGGCCGGCATCCTGG |
| Trans-KTI1 | GAGTTTCCCGTGAATACAATGA | CCCTTATCTGGGAACTACTCACA |
| Trans-KTI2 | CCTTTCCTTCCTGCCTCTCT | CCCTTATCTGGGAACTACTCACA |
| Trans-KTI3 | CATGATGATGGAACCAGGCG | CCCTTATCTGGGAACTACTCACA |
| Trans-KTI5 | GCACATGTGGGGATATTGGG | CCCTTATCTGGGAACTACTCACA |
| Trans-KTI7 | GATGGTGAAGGAAACAGGCG | CCCTTATCTGGGAACTACTCACA |
| Trans-BBI5 | CACTGCTTGAGGGTAGCACA | CCCTTATCTGGGAACTACTCACA |
| GmUBI3 | GTGTAATGTTGGATGTGTTCCC | ACACAATTGAGTTCAACACAAACCG |


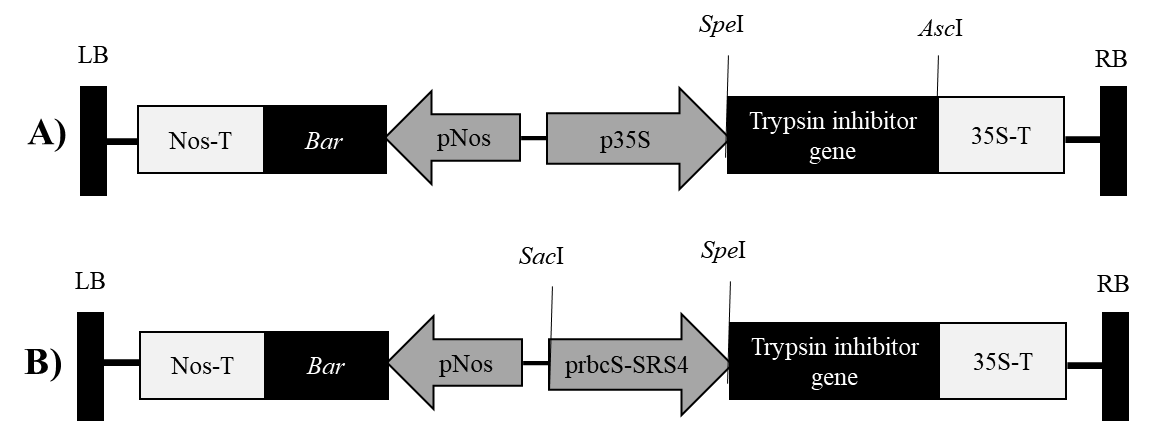


**
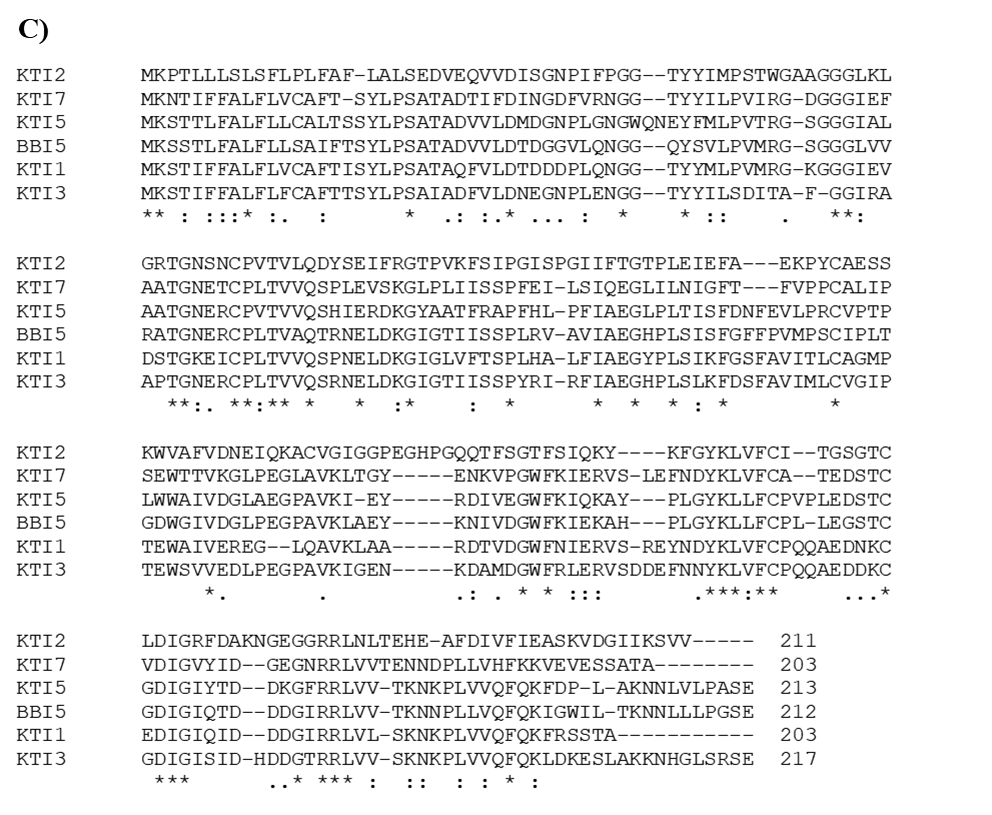
****Supplemental Figure S1:** Schematic diagrams of promoter-trypsin inhibitor gene constructs. A) & B) Left border (LB) and right border (RB) flanking the T-DNA insert are represented by black vertically elongated rectangles. p35S, 35S *Cauliflower mosaic virus* (35S CaMV) promoter; prbcS-SRS4, ribulose-1,5-bisphosphate carboxylase small subunit gene SRS4 promoter, pNOS, nopaline synthase promoter; Nos-T, nopaline synthase terminator; 35S-T, 35S CaMV terminator; *Bar*, bialaphos resistance gene; Trypsin inhibitor gene individually cloned into each vector construct were Kunitz trypsin inhibitor gene (KTI1, KTI2, KTI3, KTI5, KTI7) and Bowman-Birk inhibitor gene (BBI5). C) Protein sequence alignment of KTI2, KTI7, KTI5, BBI5, KTI1, and KTI3. Residues marked with asterisks and dots are highly conserved and semiconserved, respectively. A dash `-` denotes a gap in the alignment.


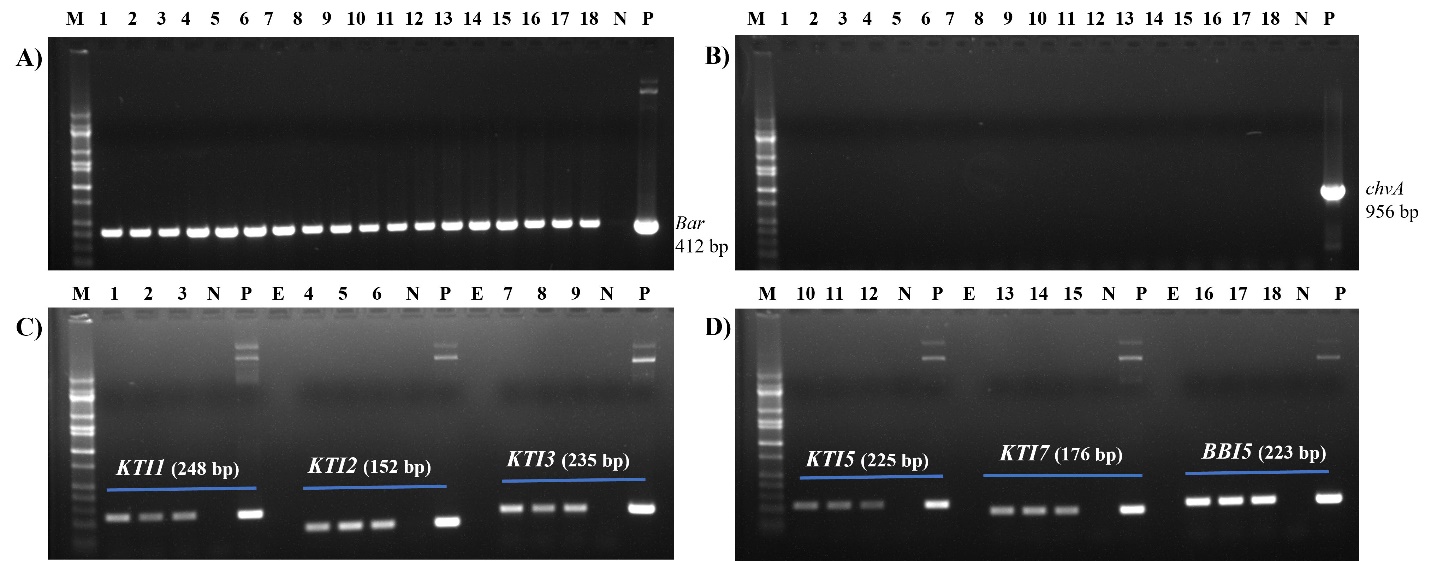


**Supplemental Figure S2:** Molecular characterization of transgenic Arabidopsis plants containing the individual 35S *Cauliflower mosaic virus* promoter-trypsin inhibitor gene (*KTI1*, *KTI2*, *KTI3*, *KTI5*, *KTI7*, *BBI5*) construct. PCR analysis of genomic DNA extracted from leaves of three-week-old plants. Lanes 1-3 are transgene *KTI1* (lines L1, L2, L3). Lanes 4-6 are transgene *KTI2* (lines L1, L2, L3). Lanes 7-9 are transgene *KTI3* (lines L1, L2, L3). Lanes 10-12 are transgene *KTI5* (lines L1, L2, L3). Lanes 13-15 are transgene *KTI7* (lines L1, L2, L3). Lanes 16-18 are transgene *BBI5* (lines L1, L2, L3). Lane N is non-transgenic wild-type Arabidopsis. Lane P is positive control template vector plasmid. Lane E is an empty well. The expected PCR amplified band size indicate presence of (A) *Bar* gene (412 bp) and (B) *chvA* gene (956 bp) (used as a control for the *Agrobacterium* contamination). No amplification was observed in transgenic lines (B). Transgene amplified fragment band for KTI1 (248 bp), KTI2 (152 bp), KTI3 (235 bp), KTI5 (225 bp), KTI7 (176 bp), and BBI5 (223 bp) was detected in transgenic lines (C) and (D). M, DNA marker.


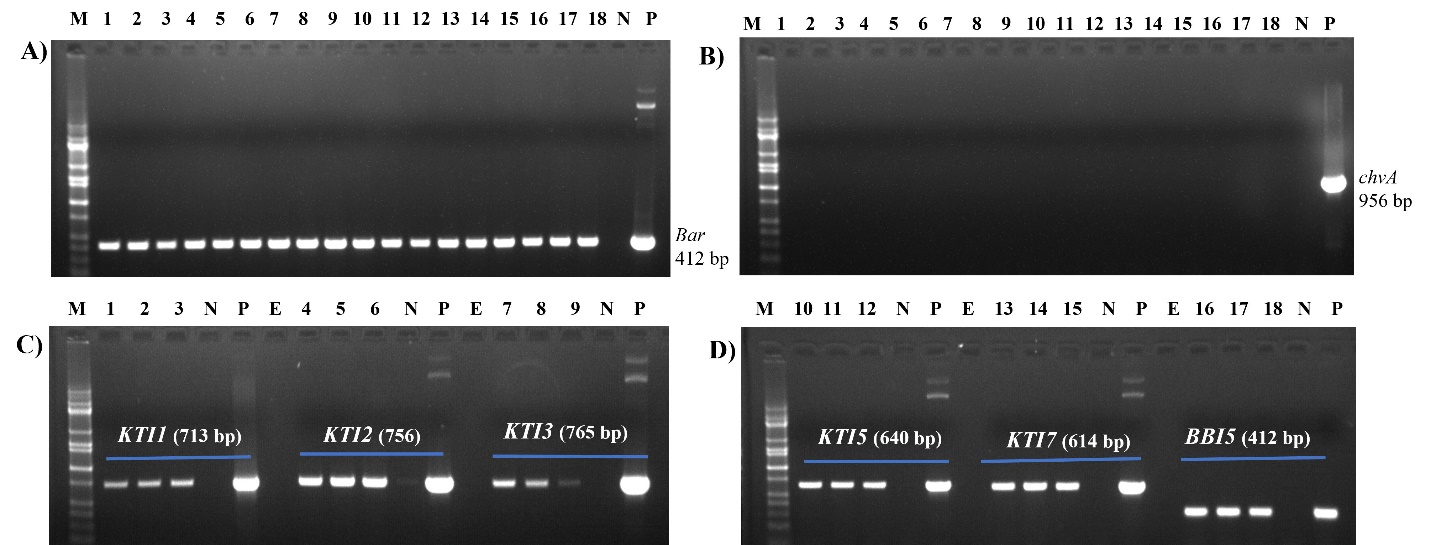


**Supplemental Figure S3:** Molecular characterization of transgenic soybean plants containing the individual 35S *Cauliflower mosaic virus* promoter-trypsin inhibitor gene (*KTI1*, *KTI2*, *KTI3*, *KTI5*, *KTI7*, *BBI5*) construct. PCR analysis of genomic DNA extracted from leaves of three-week-old plants. Lanes 1-3 are transgene *KTI1* (lines L1, L2, L3). Lanes 4-6 are transgene *KTI2* (lines L1, L2, L3). Lanes 7-9 are transgene *KTI3* (lines L1, L2, L3). Lanes 10-12 are transgene *KTI5* (lines L1, L2, L3). Lanes 13-15 are transgene *KTI7* (lines L1, L2, L3). Lanes 16-18 are transgene *BBI5* (lines L1, L2, L3). Lane N is non-transgenic wild-type soybean. Lane P is positive control template vector plasmid. Lane E is an empty well. The expected PCR amplified band size indicate presence of (A) *Bar* gene (412 bp) and (B) *chvA* gene (956 bp) (used as a control for the *Agrobacterium* contamination). No amplification was observed in transgenic lines (B). Transgene amplified fragment band for KTI1 (713 bp), KTI2 (756 bp), KTI3 (765 bp), KTI5 (640 bp), KTI7 (614 bp), and BBI5 (412 bp) was detected in transgenic lines (C) and (D). M, DNA marker.


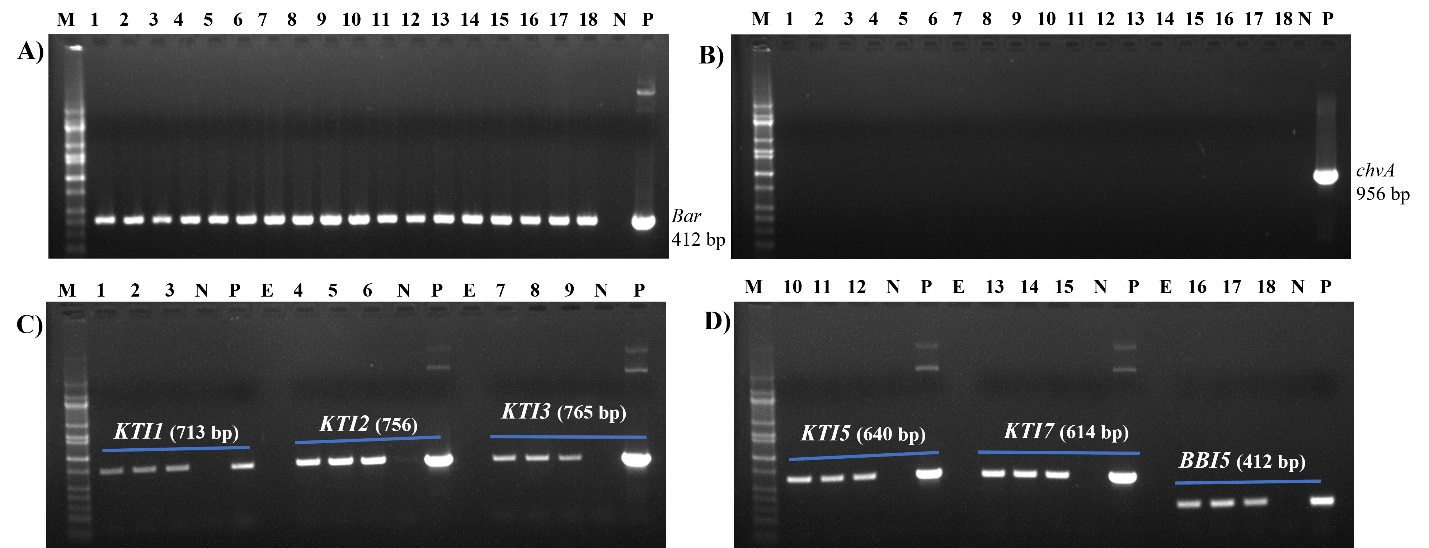


**Supplemental Figure S4:** Molecular characterization of transgenic soybean plants containing the individual rbcs-SRS4 (ribulose-1,5-bisphosphate carboxylase small subunit gene SRS4) promoter-trypsin inhibitor gene (*KTI1*, *KTI2*, *KTI3*, *KTI5*, *KTI7*, *BBI5*) construct. PCR analysis of genomic DNA extracted from leaves of three-week-old plants. Lanes 1-3 are transgene *KTI1* (lines L1, L2, L3). Lanes 4-6 are transgene *KTI2* (lines L1, L2, L3). Lanes 7-9 are transgene *KTI3* (lines L1, L2, L3). Lanes 10-12 are transgene *KTI5* (lines L1, L2, L3). Lanes 13-15 are transgene *KTI7* (lines L1, L2, L3). Lanes 16-18 are transgene *BBI5* (lines L1, L2, L3). Lane N is non-transgenic wild-type soybean. Lane P is positive control template vector plasmid. Lane E is an empty well. The expected PCR amplified band size indicate presence of (A) *Bar* gene (412 bp) and (B) *chvA* gene (956 bp) (used as a control for the *Agrobacterium* contamination). No amplification was observed in transgenic lines (B). Transgene amplified fragment band for KTI1 (713 bp), KTI2 (756 bp), KTI3 (765 bp), KTI5 (640 bp), KTI7 (614 bp), and BBI5 (412 bp) was detected in transgenic lines (C) and (D). M, DNA marker.


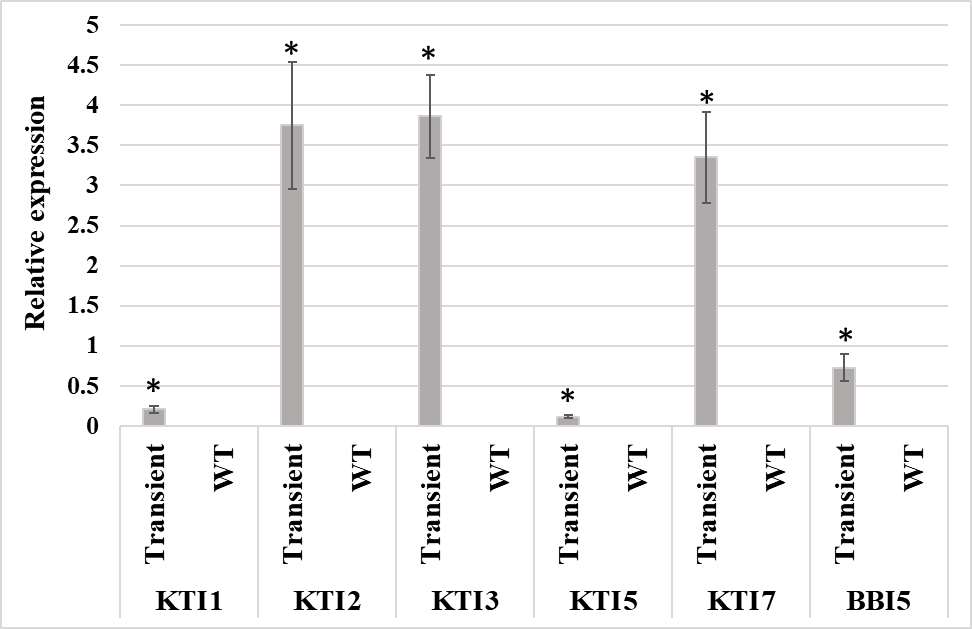


**Supplemental Figure S5:** Relative transcript levels of trypsin inhibitor genes (KTI1, KTI2, KTI3, KTI5, KTI7, BBI5) under the control of the 35S *Cauliflower mosaic virus* promoter. - Each construct was agroinfiltrated into *Nicotiana benthamiana* leaves (transient). Wild-type (WT) leaves were agroinfiltrated with a mock control solution. The relative levels of transcripts were normalized to *N. benthamiana* glyceraldehyde 3-phosphate dehydrogenase gene (GAPDH). Six independent biological replicates (plants) were used for each gene construct. Bars represent mean values of six biological replicates (plants) ± standard error. Statistical significance (p < 0.05) was determined by two-sample paired *t*-test. Bars with asterisk (*) indicate significant difference between transient and wild-type plants.


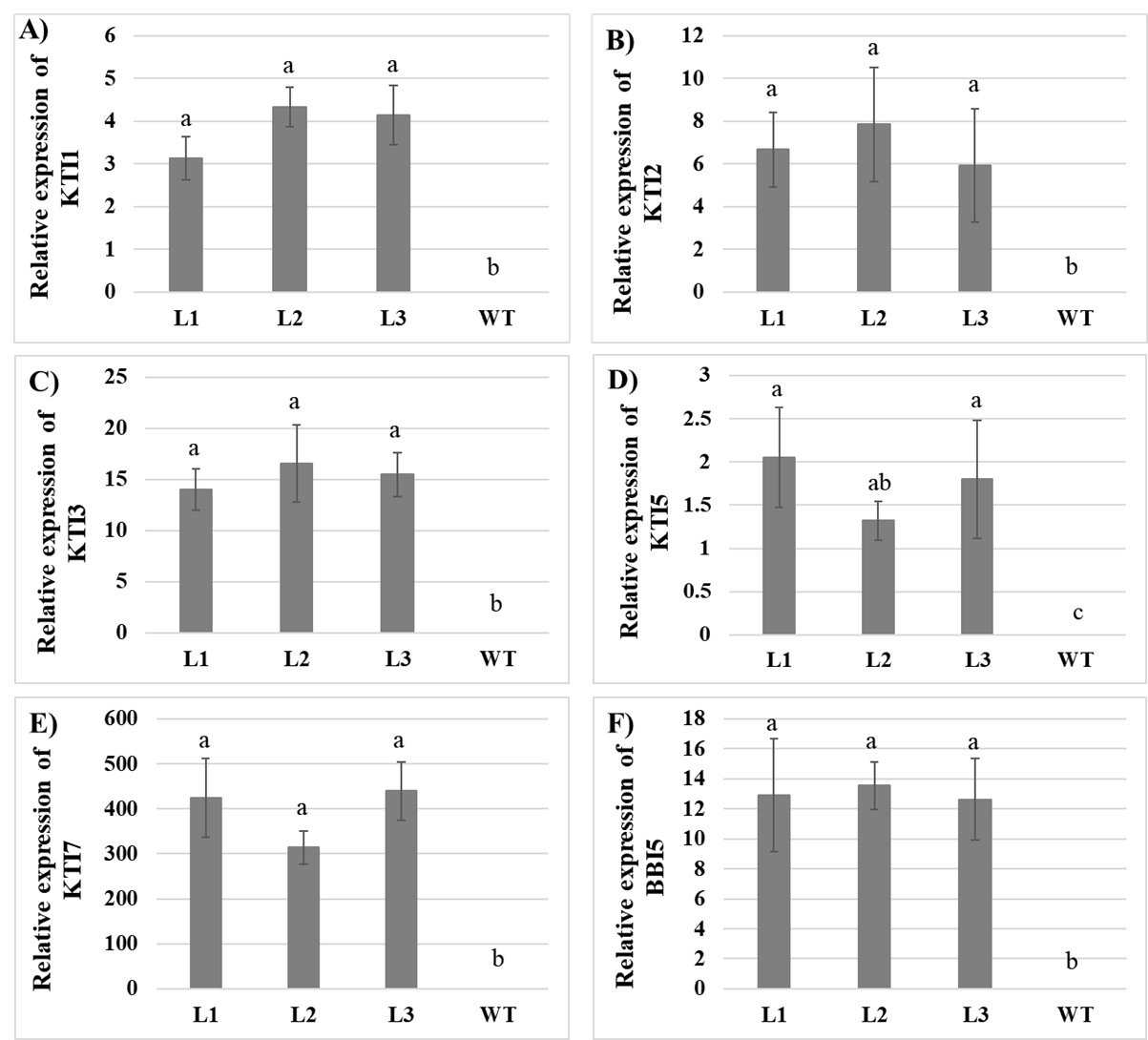


**Supplemental Figure S6:**  Expression analysis of the individual 35S *Cauliflower mosaic virus* promoter-trypsin inhibitor gene construct in leaves of six-week-old T_3_ transgenic and non-transgenic (WT) Arabidopsis plants. The relative expression of transgene (A) KTI1, (B) KTI2, (C) KTI3, (D) KTI5, (E) KTI7, and (F) BBI5. The relative levels of transcripts were normalized to Arabidopsis actin gene (*ACT*). Bars represent mean values of six biological replicates (plants) per each independent line (L1, L2, L3) ± standard error. Bars with different letters are significantly different at *p* < 0.05 as tested by one-way analysis of variance followed by a Fisher’s least significant difference.


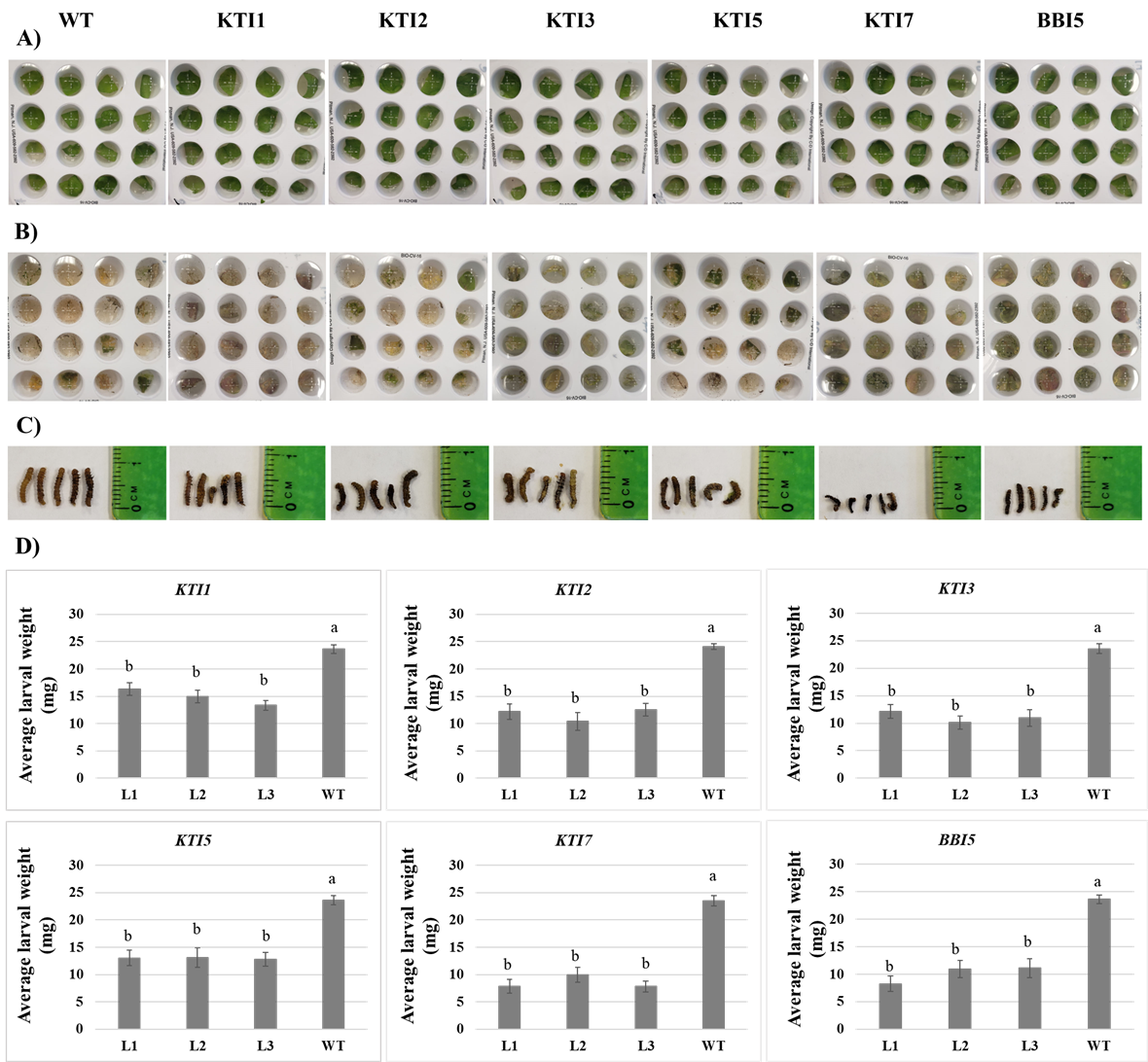


**Supplemental Figure S7:** Detached leaf-punch bioassay using six-week-old T_3_ transgenic Arabidopsis containing the individual 35S *Cauliflower mosaic virus* promoter-trypsin inhibitor gene (KTI1, KTI2, KTI3, KTI5, KTI7, and BBI5) construct and corn earworm (*Helicoverpa zea*) neonate larvae. (A) Wild-type (WT) and transgenic Arabidopsis plants detached-leaf punches before corn earworm larval inoculation. (B) Leaf punches inoculated with corn earworm neonate larvae at eight days after feeding. (C) Larval size after eight days of feeding. (D) Average larval weight after eight days of feeding. Bars represent mean values of six biological replicates (plants) per each independent line (L1, L2, L3) ± standard error. Bars with different letters are significantly different at *p* < 0.05 as tested by one-way analysis of variance followed by a Fisher’s least significant difference.


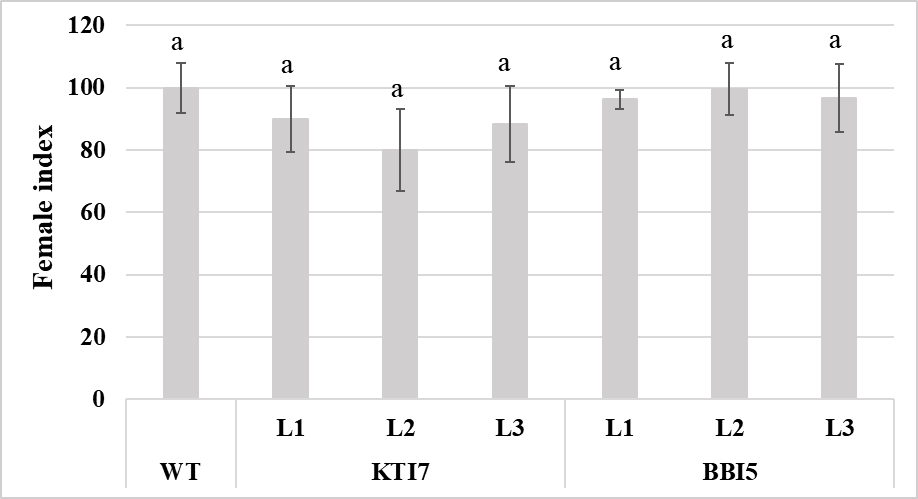


**Supplemental Figure S8:** Soybean cyst nematode (SCN, Heterodera *glycines*) bioassay of T_3_ transgenic soybean containing the individual 35S *Cauliflower mosaic virus* promoter-trypsin inhibitor gene (*KTI7* and *BBI5*) constructs. SCN HG type 0 (race 3) was used for the SCN bioassay experiments. Bars represent mean values of six biological replicates (plants) per each independent line (L1, L2, L3) ± standard error. WT, non-transgenic wild-type. Bars with same letters are not significantly different at p < 0.05 as tested by one-way analysis of variance followed by a Fisher’s least significant difference.


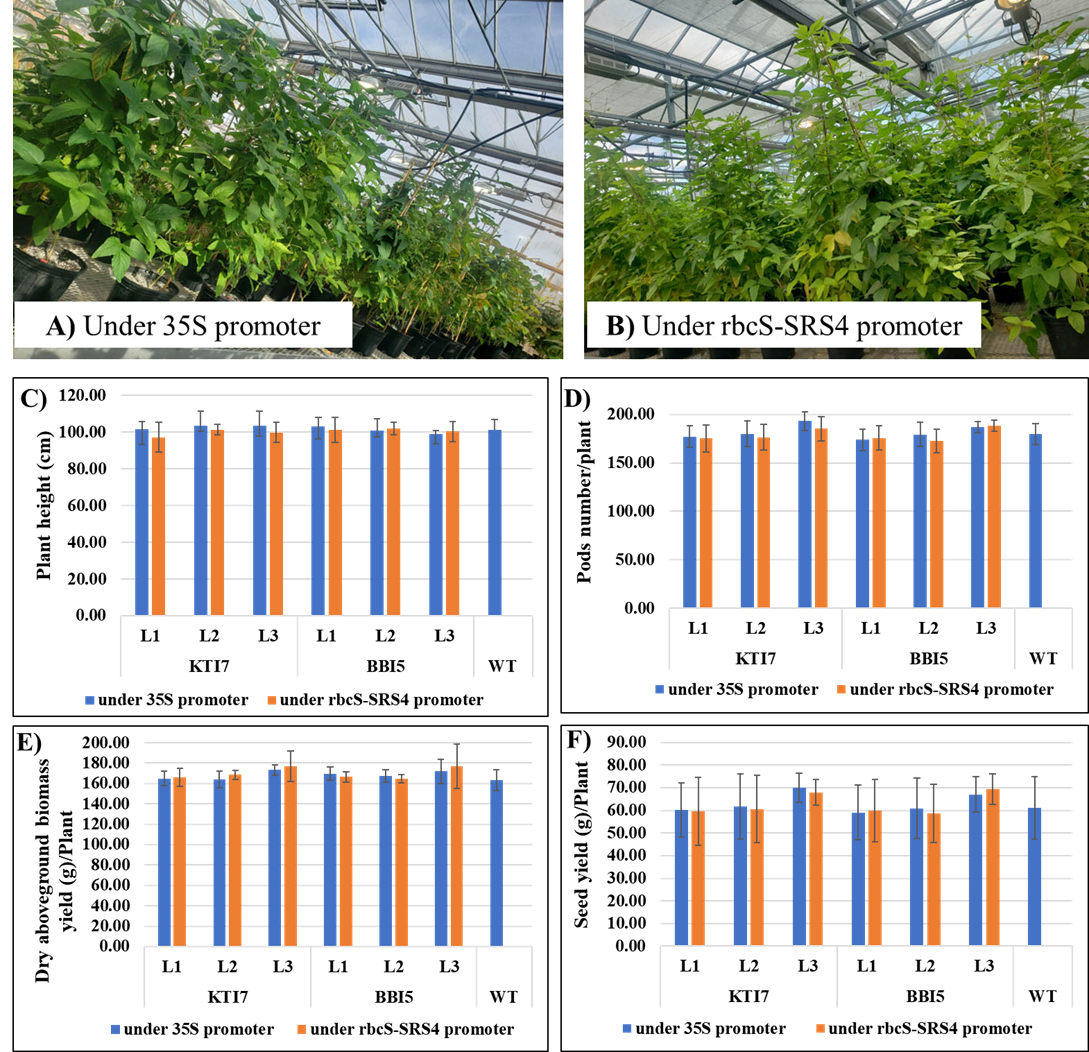


**Supplemental Figure S9:** Agronomic traits evaluation of transgenic soybean containing the individual trypsin inhibitor gene (KTI7 and BBI5) constructs and non-transgenic wild-type plants under greenhouse conditions. Representative plants with corresponding transgene under control of 35S CaMV promoter (A) or under prbcS-SRS4 promoter (B), C) Plant height, D) pods number/plant, E) dry aboveground biomass yield/plant, and F) seed yield/plant. Bars represent mean values of ten biological replicates (plants) per each independent line (L1, L2, L3) ± standard error. Statistical analysis by a two-sample paired t-test (P <0.05) indicated no significant differences between wild-type (WT) and transgenic plants.


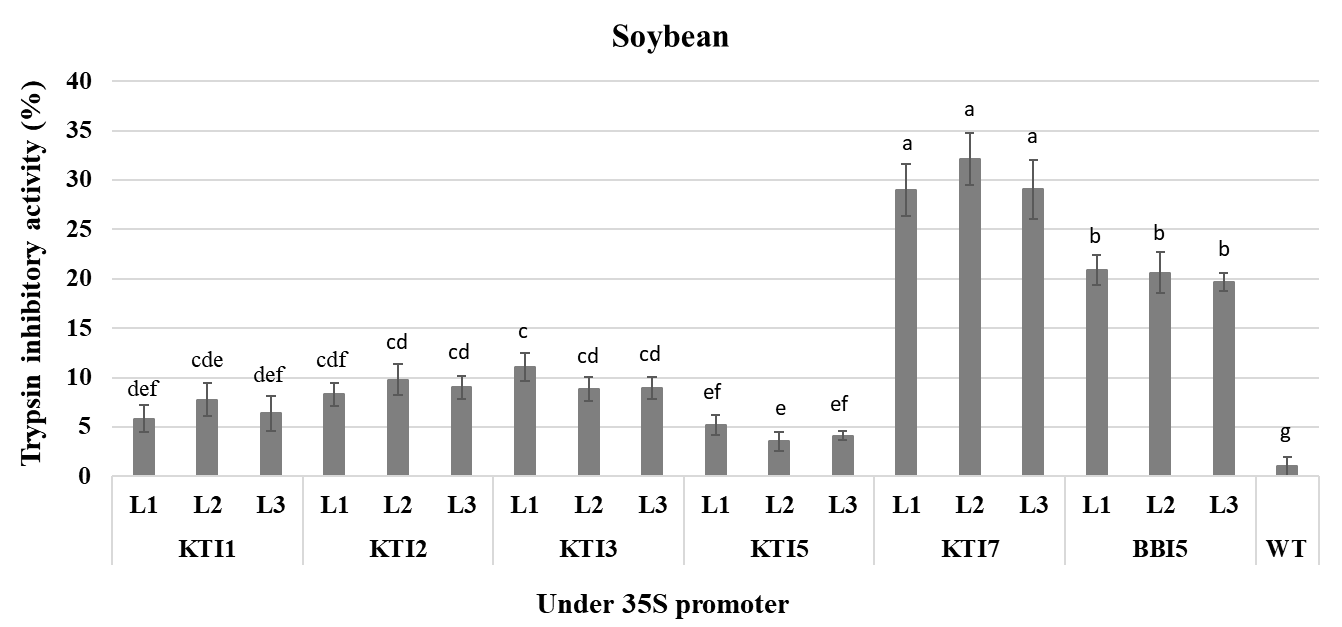


**Supplemental Figure S10:** Inhibition of gut enzyme by the individual trypsin inhibitor gene (KTI1, KTI2, KTI3, KTI5, KTI7, BBI5) construct in leaves of six-week-old T3 transgenic soybean plants under the control of 35S CaMV promoter. The percentage of trypsin inhibition activities in leaf total protein extract from transgenic plants with corresponding each type of gene relative to that of non-transgenic wild-type plants. Bars represent mean values of six biological replicates (plants) per each independent line (L1, L2, L3) ± standard error. Bars with different letters are significantly different at p < 0.05 as tested by one-way analysis of variance followed by a Fisher’s least significant difference.


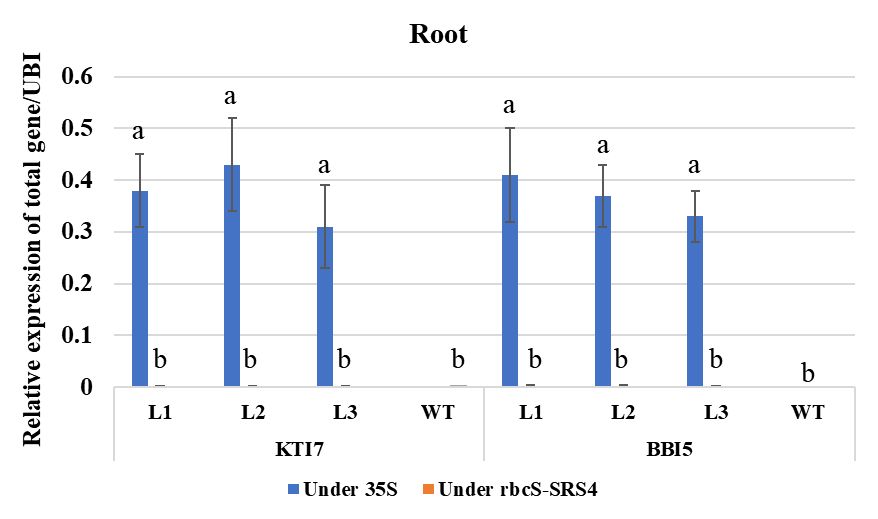


Supplemental Figure S11: Expression analysis of KTI7 and BBI5 gene construct in roots of six-week-old T3 transgenic and non-transgenic wild-type (WT) soybean plants. The relative expression of total gene corresponding to KTI7 and BBI5 under the control of 35S CaMV promoter and rbcS-SRS4 promoter. The relative levels of transcripts were normalized to soybean ubiquitin gene (GmUBI3). Bars represent mean values of six biological replicates (plants) per independent line (L1, L2, L3) ± standard error. Each gene was statistically analyzed separately. Bars with different letters are significantly different at p < 0.05 as tested by one-way analysis of variance followed by a Fisher’s least significant difference.
